# Cost and affordability of three levels of diet quality for urban households in Colombia

**DOI:** 10.1017/S1368980025000564

**Published:** 2025-06-02

**Authors:** Ana Milena Yoshioka Vargas, María del Pilar Zea León, Luis Eduardo Girón Cruz, Daniel Enrique González Gómez, Sergio A. Barona Montoya, Sara Rankin-Cortázar, Carlos Eduardo González Rodríguez

**Affiliations:** 1 Faculty of Economic and Administrative Sciences, Pontificia Universidad Javeriana Cali, Cali, Colombia; 2 Faculty of Health Sciences, Pontificia Universidad Javeriana Cali, Cali, Colombia; 3 Faculty of Engineering and Sciences, Pontificia Universidad Javeriana Cali, Cali, Colombia; 4 International Center for Tropical Agriculture (CIAT), Palmira, Colombia

**Keywords:** Least-cost diets, Healthy diets, Food prices, Food affordability, Colombia, Food systems

## Abstract

**Objective:**

To determine the minimum cost and affordability of three levels of diet quality in urban households in Cali, Colombia: a caloric-adequate diet, a nutrient-adequate diet and a recommended diet.

**Design:**

Least-cost diets were estimated for different demographic groups. The cost of caloric adequacy (CoCA) and the cost of nutrient adequacy (CoNA) were computed using linear programming models. The cost of recommended diet (CoRD) adheres to Colombia’s Food-Based Dietary Guidelines. Individualised costs were aggregated for a representative household, and affordability was assessed by comparing these costs with household food expenditures. Data sources included the National Administrative Department of Statistics, the Ministry of Health and Social Protection and the Colombia Institute of Family Welfare.

**Setting:**

Cali, Colombia.

**Participants:**

The per capita income and food expenditures of 885 urban households in Cali, taken from Colombia’s Great Integrated Household Survey.

**Results:**

The CoNA per 1000 kcal indicates that women require more nutrient-dense diets than men. Limiting nutrients include vitamin C, vitamin A, vitamin B_12_ and Ca. Three food groups: (1) meat, eggs, legumes, nuts and seeds; (2) milk and dairy products and (3) vegetables and fruits – account for about 70 % of the CoRD. The affordability analysis shows that 42·66 % of households in the 10th income percentile cannot afford the CoCA, none below the 20th percentile can afford the CoNA and only those above the 40th percentile can afford the CoRD.

**Conclusions:**

Urban households face significant barriers not only to affording diets that promote long-term health but also to those that meet nutritional requirements.

The latest report on The State of Food Security and Nutrition in the World confirms that the challenges of ending hunger, food insecurity and all forms of malnutrition – especially stunting, undernutrition, micronutrient deficiencies, overweight and obesity – continue to emerge^(1)^. Although malnutrition in the population is the result of various factors, it is often fundamentally related to inadequate intake of essential nutrients due to limited income^(2,3)^. Recently, several factors have contributed to exacerbating the lack of access to sufficient and nutritious food: global health emergencies such as COVID-19^(4)^; geopolitical events like the Russia-Ukraine conflict^(5)^ and climate change, along with climate-related shocks. Additionally, high inflation rates, particularly affecting food prices^(6)^, disproportionally impact Cali’s poorer households, which already allocate a larger share of their budgets to food (Engel’s Law) and are primarily net buyers^(7,8)^, making them especially vulnerable to fluctuations in food prices.

Recent studies propose assessing economic access to sufficient and nutritious food by measuring affordability across different levels of diet quality at a minimum cost. Commonly used metrics include (i) the cost of caloric adequacy (CoCA), which is the minimum cost of a diet that provides adequate calories to cover the individual’s daily estimated energy requirement (EER)^(9,10)^; (ii) the cost of nutrient adequacy (CoNA), which is a least-cost nutrient-adequate diet that, in addition to satisfying the EER, provides adequate levels of macro- and micronutrients within their minimum and maximum limits to prevent deficiencies and avoid toxicity^(11–15)^ and (iii) the cost of a recommended diet (CoRD) or a healthy diet, which is a diverse least-cost diet that adheres to the food group recommendations outlined in Colombia’s national Food-Based Dietary Guidelines (FBDG)^(16,17)^.

A prevalent trend in the literature is to estimate the minimum cost of various diets for specific populations, typically a median healthy woman of reproductive age^(2,12,18,19)^. To the best of our knowledge, except for a recent global study on CoNA estimates^(20)^, there has been limited research on how demographic factors influence these cost estimates. Similarly, affordability analyses of least-cost diets predominantly use representative units and individualised diets, which specify dietary composition for each individual or representative household member^(18,19,21,22)^. More recently, a new approach has emerged for assessing the affordability of shared diets. These diets are optimised to be shared among household members, with individual quantities adjusted to each member’s energy requirements while considering the combined nutrient requirements of all household members, accounting for the nutrient requirements of the neediest member^(23)^.

Most studies examining one or two of the three least-cost diet metrics have been conducted primarily in South Asia^(10,15,16,21)^, Southeast Asia^(9,24)^ and East Africa^(23,25)^, with fewer focusing on Latin America^(15,22)^. Their findings are not easily applicable to Latin American contexts, such as Colombia’s, or to local city contexts with high levels of economic inequality, such as Cali (Gini: 0·512), whose food system is characterised by a lack of agricultural vocation, except for small-scale production and sugarcane crops. Furthermore, the city’s supply system has demonstrated vulnerability to exogenous events such as climatic and environmental shocks and social disturbances^(26)^, highlighting its dependence on stable market conditions. This is evidenced by the 2021 Colombian protests during the COVID-19 pandemic, which were triggered by a failed tax-reform proposal and led to supply-chain disruptions and shortages of basic goods, including food.

In this context, using data for September 2022 gathered from Cali’s urban population, this study aims to determine the minimum cost and affordability of caloric-adequate, nutrient-adequate and healthy diets for urban households in the city. First, employing a differentiated approach, the study estimates the minimum cost of these least-cost diets according to age, sex and physiological condition. Second, our study performs an affordability analysis by adopting a representative household approach with individualised diets. This analysis includes calculating two measures: the ratio of per capita diet costs to average household per capita food expenditure and the proportion of households unable to afford either of the least-cost diets.

## Data and methods

### Data

#### Retail prices of locally available foods and their nutrient composition

Data on wholesale prices and supply were obtained from the Information System for Prices and Supply of the Agricultural Sector (SIPSA)^(27)^, which captures information from the Valle del Cauca Supply Centre (CAVASA) and four wholesale satellite marketplaces in Cali. The locally available foods were those with a monthly supply in kilograms above the first quartile of the distribution, reflecting seasonal supply patterns. Food items with very low caloric intake, such as condiments, herbs, spices and extracts, were excluded.

Based on data from a large local supermarket for the third quarter of 2022, retail prices of locally available foods were calculated by applying the retailer’s mark-up to wholesale prices, with mark-ups assumed to be constant for foods within the same category. Finally, retail prices were expressed per 100 grams of the edible portion (see online supplementary material, Supplemental Table S1).

Energy and nutrient content of the foods consumed by the country’s population was assigned according to the Colombian Food Composition Table^(28)^. The nutrient composition included macronutrients (proteins, lipids and carbohydrates) and thirteen micronutrients (Ca, Zn, Fe, Mg, phosphorus, vitamin C, thiamine, riboflavin, niacin, folate, vitamin B_12_, vitamin A and Na). Information gaps for certain nutrients were addressed through food homologation, imputation by similarity using the most reliable available sources or by borrowing data from the sole available source^(29)^.

#### Estimated energy and essential nutrient requirements of Cali’s urban population

The EER was calculated based on information about population-specific characteristics, such as average weight, height and predominant physical activity levels for each age group and sex in the region. For individuals under 65 years of age, these characteristics were obtained from the National Survey of Nutritional Situation in Colombia (ENSIN 2015)^(30)^. For those aged 65 years and older, information was sourced from Colombia’s Health, Well-being and Ageing Study (SABE)^(31)^. EER for children was estimated under Resolution 3803 of 2016 of the Colombian Ministry of Health and Social Protection, which established the Recommendations for Energy and Nutrient Intake (RIEN)^(32)^. To account for population-specific characteristics, equations described in the dietary reference intakes by the Institute of Medicine^(33)^ were used to estimate adults’ EER. For pregnant and lactating women, additional caloric needs during the third trimester of pregnancy and the first six months (postpartum) of exclusive breastfeeding were added to the baseline requirements for non-pregnant and non-lactating women of the same age group. Finally, to aggregate across age groups, a weighted average of the EER was computed based on the projections of the National Population and Housing Census, with weights reflecting the population distribution by sex and age.

To establish the minimum and maximum macronutrient requirements by age group, the acceptable macronutrient distribution range was used. For micronutrients, the lower limit corresponded to the Estimated Average Requirement (EAR) that meets the requirements of 50 % of healthy individuals, while the tolerable upper intake level represented the highest intake level needed to avoid the risk of adverse health effects for the majority of the population. For Na, Adequate Intake (AI) was considered as the minimum reference value and the upper intake level as the maximum. For nutrients without an upper intake level – namely, riboflavin, thiamine and vitamin B_12_ – only the estimated average requirement was used.

### Estimation of the minimum costs of diets

#### Cost of a caloric-adequate diet

The CoCA is estimated by selecting the food – or set of foods – that provides the number of calories necessary to satisfy the EER. Based on prior research^(3,9)^, in a given demographic group 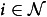 , the CoCA has been calculated using the following linear programming model:

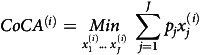

subject to

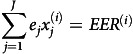




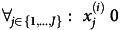

where 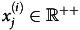 is the quantity – expressed in grams – of food 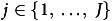 for an individual in the *i*-th demographic group; 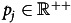 and 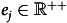 correspond to the retail price and energy content of the *j*-th food, respectively. The EER for an individual in demographic group 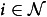 is expressed as 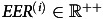 .

The optimal solution to the linear programming model is to construct a diet composed of food whose price-per-kilocalorie is minimal. Thus, the optimal solution follows the form 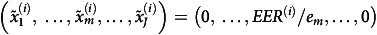 , where for some 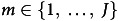 , 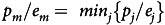 . This means selecting the quantity of the least-cost starchy staple food needed to meet the EER.

#### Cost of nutrient-adequate diet

The CoNA is determined by the set of available foods that, for the location and period of study, satisfy the EER at the lowest cost, as well as the lower and upper limits of macro- and micronutrient intake. For a demographic group 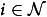 , following extensions of Stigler’s seminal article^(34)^, the CoNA metric is obtained as the solution to the following linear programming model

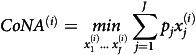

subject to

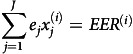




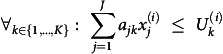




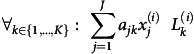




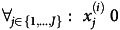




Given a demographic group, 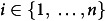 , and a nutrient, 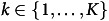 , 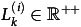 and 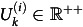 express the lower and upper limits of the *k*-th nutrient for the *i*-th demographic group, respectively; for all 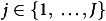 , 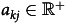 corresponds to the content of the *k*-th nutrient in the *j*-th locally available food.

This linear programming model facilitates the identification of limiting nutrients, which are those potentially at low levels^(25,35)^. Formally, a nutrient 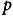 is considered limiting if, given the optimal solution 
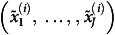
 , its intake level exactly meets the lower limit, i.e. 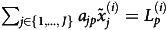 .

#### Cost of recommended diet

The CoRD is determined by the lowest-cost set of foods that meet the recommendations for food groups specified in the FBDG for the Colombian population^(36)^. These guidelines provide the required number of food exchanges for various demographic groups, adjusted for both EER by age group and caloric intake by food group (see online supplementary material, Supplemental Table S2).

Calculating the CoRD is simpler than estimating the CoNA. It involves straightforward methods such as selecting the least-cost food items from various food groups. The number selected from each group is determined by two criteria: (i) at least one item per food group to ensure intergroup diversity and (ii) 2–4 items per group, when the recommendation exceeds two exchanges, to ensure intragroup diversity^(16,18)^. Consequently, our study selected 12 least-cost items, as detailed in Table 1. These items were chosen based on their price per edible serving, calculated by dividing the price per 100 grams of the edible portion by 100 and multiplying by the serving size (in grams) specified in the dietary guidelines.

**Table 1.** Foods selected by food group or subgroup based on Colombia’s food-based dietary guidelines (GABA)

GABA Group	GABA Subgroup	No. of Foods
Cereals, roots, tubers, and bananas	Cereals	3
	Roots	
	Tubers	
	Bananas	
Fruits and vegetables	Vegetables	2
	Fruit	2
Milk and dairy products	Milk	1
	Dairy products (cheeses, yogurt, and kumis*)	
Meats, eggs, legumes, nuts, and seeds	Meats	2
	Eggs	
	Dried legumes and vegetable mixes	
	Nuts and seeds	
Fats	Polyunsaturated	1
	Monounsaturated	
	Saturated	
Sugars	Simple sugars	1

GABA = Food-Based Dietary Guidelines. Source: Own calculations based on information from GABA. *Kumis = traditional fermented cow’s milk drink.

After selecting the twelve least-cost food items within each food group, the quantity of each item in the healthy diet adheres to two criteria: (i) all items within a group are included in the same quantity and (ii) each group must provide the number of food exchanges specified by the dietary recommendations for each demographic group, as outlined in online supplementary material, Supplemental Table S2. It is straightforward to show that these criteria align with established methods for estimating the CoRD, as described in prior research^(16,17)^, which defines the CoRD as the average price per edible serving for each food group multiplied by the number of servings recommended for that group.

### Affordability indicators

The affordability of least-cost diets for 885 urban households, based on the Great Integrated Household Survey and using expansion factors, was assessed through a representative household approach with individualised diets. This means the total minimum cost of a diet for each household was calculated as the sum of the estimated minimum cost for each member. According to the Great Integrated Household Survey published in September 2022, the average urban household size in Cali has approximately three members, with 72 % of these being nuclear households^(37)^. By verifying household composition by age, the representative household is defined as follows: a moderately active woman and man aged 31–50 years and a moderately active girl aged 9–13 years.

For urban households in Cali, the distribution of monthly per capita income was derived from the September 2022 Great Integrated Household Survey, and the share of household food expenditure was calculated using data from the 2022 Quality-of-Life Survey, conducted annually. Following Engel’s law, this share is calculated across various income levels, specifically for each percentile of the per capita household income distribution (Table 2). The positively skewed distribution indicates a significant concentration of households at lower income levels, suggesting potential economic limitations in access to food for a substantial proportion of urban households.

**Table 2.** Current income and monthly food expenditure per capita by household by percentiles of per capita income

Percentiles	Per Capita Income (USD)			Per Capita Food Expenditure (USD)			Proportion of Food Expenditure (%)
	Mean	sd	Maximum	Mean	sd	Maximum	
10	53·09	25·66	88·31	20·70	10·01	34·44	39 %
20	106·47	9·13	121·92	41·52	3·56	47·55	39 %
30	142·61	11·57	163·96	51·34	4·16	59·03	36 %
40	186·69	11·95	206·08	67·21	4·30	74·19	36 %
50	226·19	12·11	248·42	79·17	4·24	86·95	35 %
60	270·00	15·10	299·43	94·50	5·28	104·80	35 %
70	333·53	21·06	379·86	106·73	6·74	121·55	32 %
80	432·18	35·77	494·42	138·30	11·45	158·22	32 %
90	609·27	74·99	738·44	158·41	19·50	191·99	26 %
100	1346·45	734·07	5284·02	350·08	190·86	1373·84	26 %

Source: Own calculations based on information from the Great Integrated Household Survey (GIHS) and Quality-of-Life Survey (QLS).

Two measures of affordability are computed at each income level: first, the ratio of the estimated per capita minimum cost of a diet for the representative household to the average per capita household expenditure on food; and second, the proportion of households unable to afford each least-cost diet. If the cost-to-expense ratio is 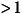 , it reveals how many times the diet is more expensive than the average per capita food expenditure, while a ratio of 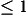 indicates the proportion of the average per capita food expenditure required to access the least-cost diet.

## Results

### Estimated minimum cost of diet

### Estimated daily cost of caloric adequacy

**Figure 1.**
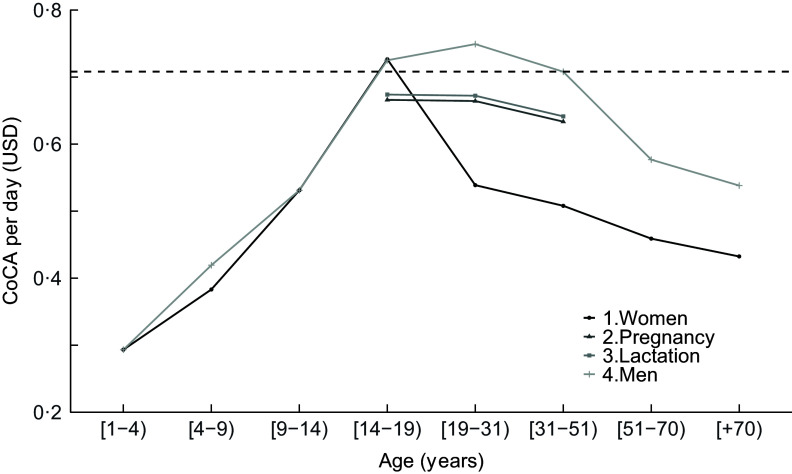
Cost of caloric adequacy per day. The daily CoCA (USD) estimates were differentiated by age, sex, and physiological condition. The dashed line represents the weighted average daily CoCA (0·71 USD (2680·5 COP)). Source: Own calculations based on the information of the study.

The linear programming model used to estimate the CoCA metric indicates that the optimal solution is the quantity of the cheapest starchy staple food – in this case, a variety of rice priced at 0·31 USD (1192·07 COP) per 1000 kcal – that meets the EER. This price and the individuals’ EER together directly determine the daily CoCA results. Our findings reveal that the trajectory of the estimated CoCA was similar for women and men aged 1–18 years. For women, the maximum CoCA value was reached in the 14–18 age group at 0·87 USD (3278·2 COP), and for men, the maximum CoCA value was reached in the 19–30 age group at 0·89 USD (3379·8 COP). The minimum CoCA value was found in the 1–3 age group for both sexes. The estimated CoCA of pregnant women and lactating mothers was lower compared with that of men and was significantly higher for men than for women in the 18–50 age group (Figure 1 and see online supplementary material, Supplemental Table S3).

#### Estimated cost of nutrient adequacy

##### Estimated daily cost of nutrient adequacy and cost of nutrient adequacy per 1000 kcal

**Figure 2.**
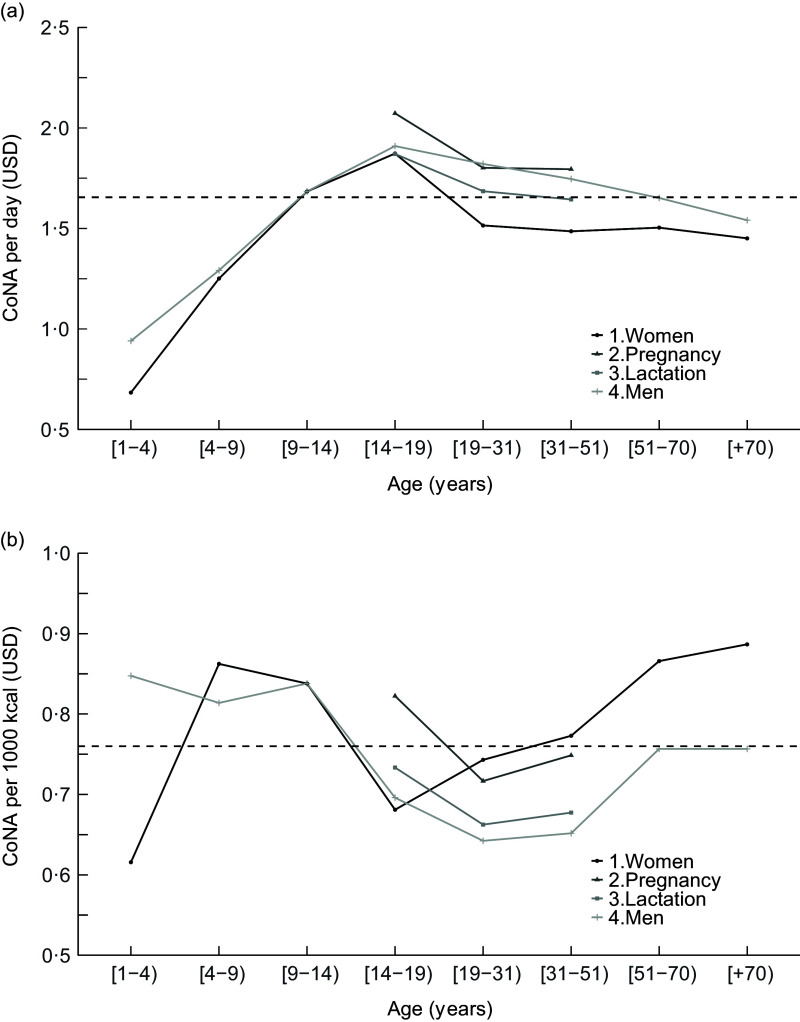
Cost per day and cost per 1000 kcal for a nutrient-adequate diet. The CoNA estimates were differentiated by age, sex, and physiological condition. (a) Estimated daily CoNA (USD). (b) Estimated CoNA per 1000 kcal. (USD) The dashed line represents (a) the weighted average daily CoNA (1·66 USD (6266·16 COP)) and (b) the average CoNA per 1000 kcal (0·76 USD (2861·1 COP)). Source: Own calculations based on the information of the study.

The trajectory of the daily CoNA was similar for women and men aged 4–18 years. In the 14–18 age group, the estimated daily CoNA reached its maximum value for men at 1·91 USD (7228·2 COP) and women at 1·87 USD (7088·3 COP). Among pregnant women and lactating mothers, the lowest daily CoNA was found in lactating mothers aged 31–50 years (1·64 USD (6223·2 COP)), and the highest daily CoNA was found in pregnant women under 18 years of age (2·07 USD (7845·5 COP)) (Figure 2(a); for values, see online supplementary material, Supplemental Table S4).

In contrast to the daily CoNA, the CoNA per 1000 kcal was calculated to adjust for differences across energy requirements. The median CoNA per 1000 kcal was calculated to be 0·75 USD (interquartile range: 0·68–0·83). The highest levels were observed in girls aged 4–8 years and women aged 51–69 years (Figure 2(b); for values, see online supplementary material, Supplemental Table S4). Notably, among individuals aged 18 years and older, women had a higher CoNA per 1000 kcal than men, indicating a greater need for a nutrient-dense diet. In addition, younger individuals aged 4–13 years and adults aged 31 years and older exhibited a markedly higher CoNA per 1000 kcal compared with other age groups.

##### Limiting nutrients

Estimating the CoNA through a linear programming model makes it possible to identify limiting nutrients – i.e. those that significantly constrain the estimated minimum cost of the diet. This study reveals that, across demographic groups, vitamin C, vitamin A, vitamin B_12_ and Ca are the vitamins and minerals that significantly constrain the estimated CoNA (Table 3). An analysis of specific demographic groups reveals that CoNA is highly sensitive to Fe requirements in pregnant women. Zn is particularly costly for men, except those aged 9–13 years, and for girls aged 8 years or younger, as well as those aged 51 years or older. Additionally, Zn requirements also restrict estimated CoNA for lactating women over 18 years. Similarly, Mg influences CoNA for individuals aged 8 years or younger and those aged 31 years or older, regardless of sex.

**Table 3.** Limiting nutrients in the least-cost nutritious diet

	Age (years)	Protein (%)	Lipids (%)	Carbohydrates (%)	Vitamin C (%)	Folates (%)	Vitamin A (%)	Thiamine (%)	Riboflavin (%)	Niacin (%)	Vitamin B_12_ (%)	Mg (%)	Phosphorus (%)	Sodium (%)	Ca (%)	Fe (%)	Zn (%)
Women	(1–4)	0	0	18·8	0	111·6	0	185·6	132·9	61·9	0	0	0·5	10·1	0	73·1	0
	(4–9)	0	0	28·1	0	50	0	0	137	61·9	0	0	60·8	0	0	89·1	0
	(9–14)	14·7	0	23·9	0	70·5	0	32·5	101·7	36	0	4·6	0	0	0	87·4	12·6
	(14–19)	0	0	26·6	0	67·6	0	41·7	58·3	34·2	0	0	9	0	0	75·7	17
	(19–31)	0	0	26·4	0	103·3	0	65·9	43·3	44·6	0	10·5	96·2	0	0	66·5	19
	(31–51)	0	0	26·6	0	87·2	0	50·4	42·2	39·5	0	0	86·1	0	0	58·7	8·9
	(51–70)	0	0	27·4	0	51·1	0	31·6	69·5	10·2	0	0	87·8	0	0	105	0
	≥ 70	1·8	0	26·9	0	44·5	0	30·9	73	0	0	0	86·8	0	0	87·4	0
Pregnancy	**<** 18	21·1	0	6·1	0	53·9	0	42	60·5	19·5	0	4	67·4	0	0	0	56
	(19–31)	0	0	24·5	0	92·3	0	66	55·1	36·4	0	20·7	140·9	0	0	0	29·8
	(31–51)	3·3	0	22·3	0	65·5	0	23·1	35·1	11·6	0	16·7	148·4	0	0	0	35
Lactation	**<** 18	0	0	16·4	0	71·8	0	53·8	21·6	35·7	0	16·6	36·1	0	0	113·7	6·6
	(19–31)	0	0	26·1	0	105·8	0	111·5	28·4	69·2	0	37·2	132	0	0	148·5	0
	(31–51)	0	0	25·8	0	88·3	0	78·8	12	49·2	0	32·1	128·1	0	0	133·5	0
Men	(1–4)	0	0	18·3	0	90·9	0	38·9	133·8	91·7	0	0	14·7	0	0	115·7	0
	(4–9)	0	0	28·2	0	81·7	0	40·2	145·8	91·1	0	0	60·1	0	0	113·5	0
	(9–14)	14·6	0	23·9	0	70·5	0	32·5	101·7	36	0	4·6	0	0	0	78·9	12·6
	(14–19)	13·1	0	22·8	0	115·9	0	78·5	61·3	24·9	0	2·9	33·1	0	0	75·6	0
	(19–31)	0	0	27	0	212·5	0	211·9	90·1	131·7	0	6·1	152·5	0	0	190·1	0
	(31–51)	0	0	26·5	0	212·5	0	199·2	76	110	0	0	136·4	0	0	190·7	0
	(51–70)	4·2	0	24·7	0	133	0	91·6	55·3	20·4	0	0	143·8	0	0	106·6	0
	≥ 70	4·2	0	24·7	0	117·4	0	78·8	44·9	12·3	0	0	127·5	1·1	0	92·7	0

For any nutrient, the difference (%) between the optimal CoNA contribution and the minimum level of intake needed for the nutrient considered is reported. Source: Own calculations.

##### Estimated daily cost of recommended diet

**Figure 3.**
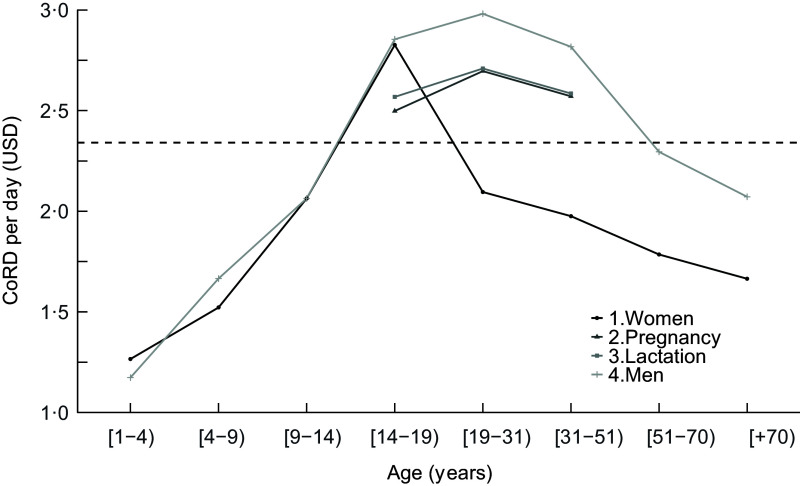
Cost per day of a recommended diet. The CoRD estimates were differentiated by age, sex, and physiological condition. The dashed line represents the weighted average CoRD (2·34 USD (8860·11 COP)). Source: Own calculations based on the information of the study.

Figure 3 shows that for women and girls, the highest CoRD was found in adolescents aged 14–18 years (2·83 USD (10 697·40 COP)), while for men and boys, it was found in adults between 19–30 years (2·98 USD (11 284·4 COP)). Among pregnant women and lactating mothers, the lowest CoRD was registered among pregnant women under 18 years of age (2·5 USD (9455·5 COP)), while the highest CoRD was registered among lactating mothers aged 19–30 years (2·71 USD (10 254·8 COP)). Greater variations in the CoRD can be seen across age groups, and the difference in the minimum costs between men and women in the 19–30 age group (2·98 USD (11 284·38 COP) *v*. 2·10 USD (7931·35 COP)) and the 31–50 age group (2·82 USD (10 663·4 COP) *v*. 1·98 USD (7477·8 COP)) are noticeable. (For details, see online supplementary material, Supplemental Table S5.)

### Affordability of a caloric-adequate diet, a nutrient-adequate diet and a healthy diet

#### Indicator 1: Affordability for urban households

**Figure 4.**
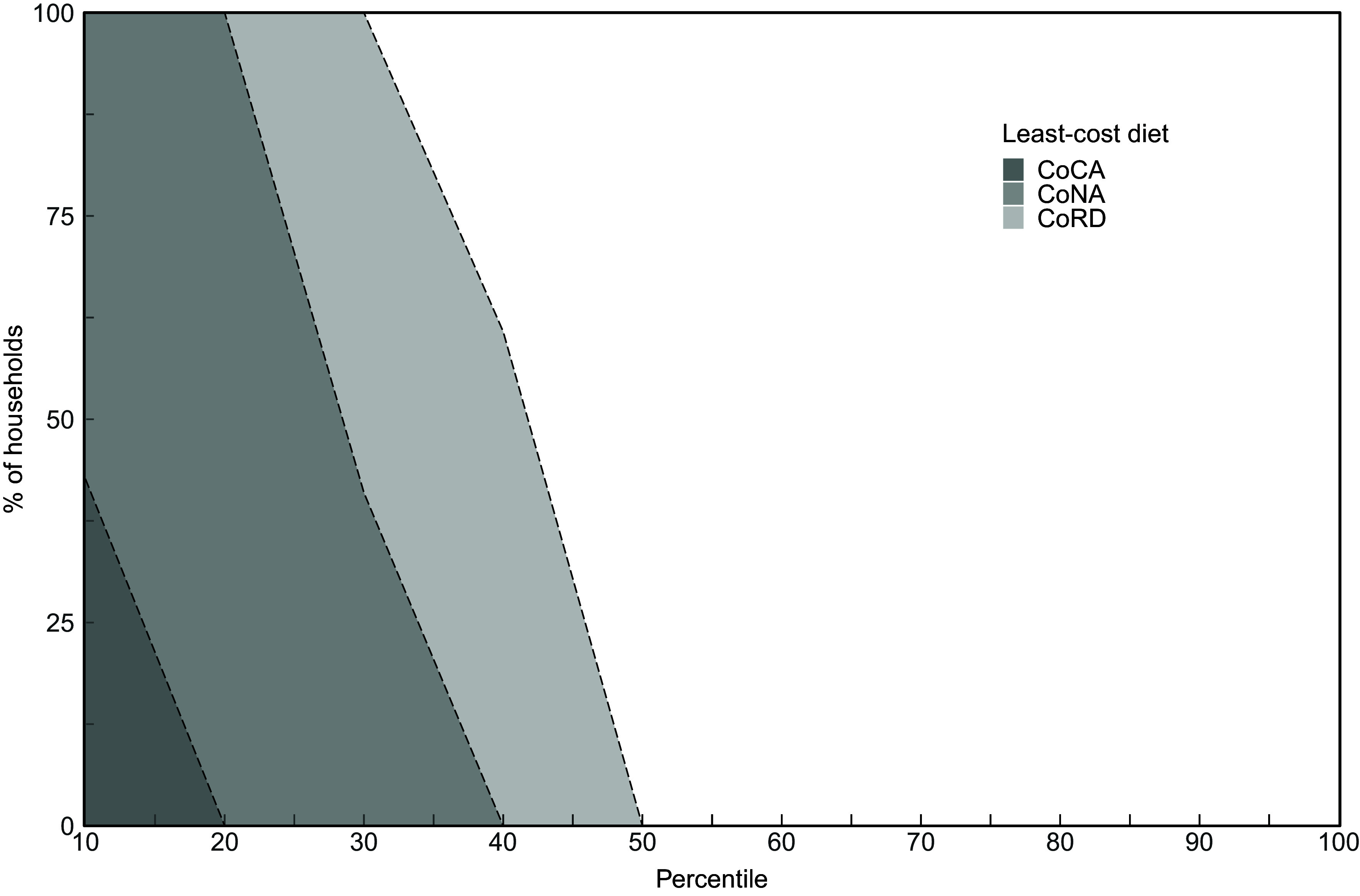
Proportion (%) of urban households in Cali, Colombia, that cannot afford any of the three diet types. The proportion of households, differentiated by percentiles of per capita income, whose per capita expenditure on food is lower than the per capita cost of each diet type. The white area represents the proportion of households (63·92 %) that can afford the three diet types. Source: Own calculations based on the information of the study.

Using the approach of individualised diets for a representative household, Table 4 presents the minimum daily costs for three levels of increasing diet quality. These costs are differentiated by the age group and sex of each household member and include the CoRD:CoCA and CoRD:CoNA ratios. The minimum costs associated with each type of diet and the information on the monthly per capita food expenditure per urban household make it possible to identify significant economic barriers to achieving certain dimensions of food security^(17)^. Approximately 42·66 % of households in the 10th percentile cannot afford the CoCA, accounting for 4·27 % of all urban households in the city. Additionally, in the 10th–20th percentiles, 100 % of households cannot afford the CoNA. In the 30th percentile, 40·97 % cannot afford the CoNA. Thus, approximately 24·10 % of households cannot afford a diet that provides the necessary levels of calories and essential nutrients. Lastly, all households in the 10th–30th percentiles cannot afford the CoRD, while approximately 39·23 % of households in the 40th percentile can afford this type of diet. Approximately 36·08 % of urban households in Cali cannot afford a diverse diet that not only meets the national dietary recommendations but also promotes long-term health (Figure 4).

**Table 4.** Least-cost diet by three increasing levels of diet quality for representative households

Age (years)	Sex	CoCA	CoNA	CoRD	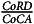	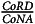
(31–51)	Men	0·84	1·75	2·82	3·34	1·61
(31–51)	Women	0·61	1·49	1·98	3·26	1·33
(9–14)	Women	0·63	1·68	2·06	3·26	1·23
Total Cost (Household)		2·08	4·92	6·86	3·29	1·39
Cost Per Capita		0·69	1·64	2·29	3·29	1·39

Source: Own calculations based on the information of the study.

#### Indicator 2: Cost-to-expense ratio

**Figure 5.**
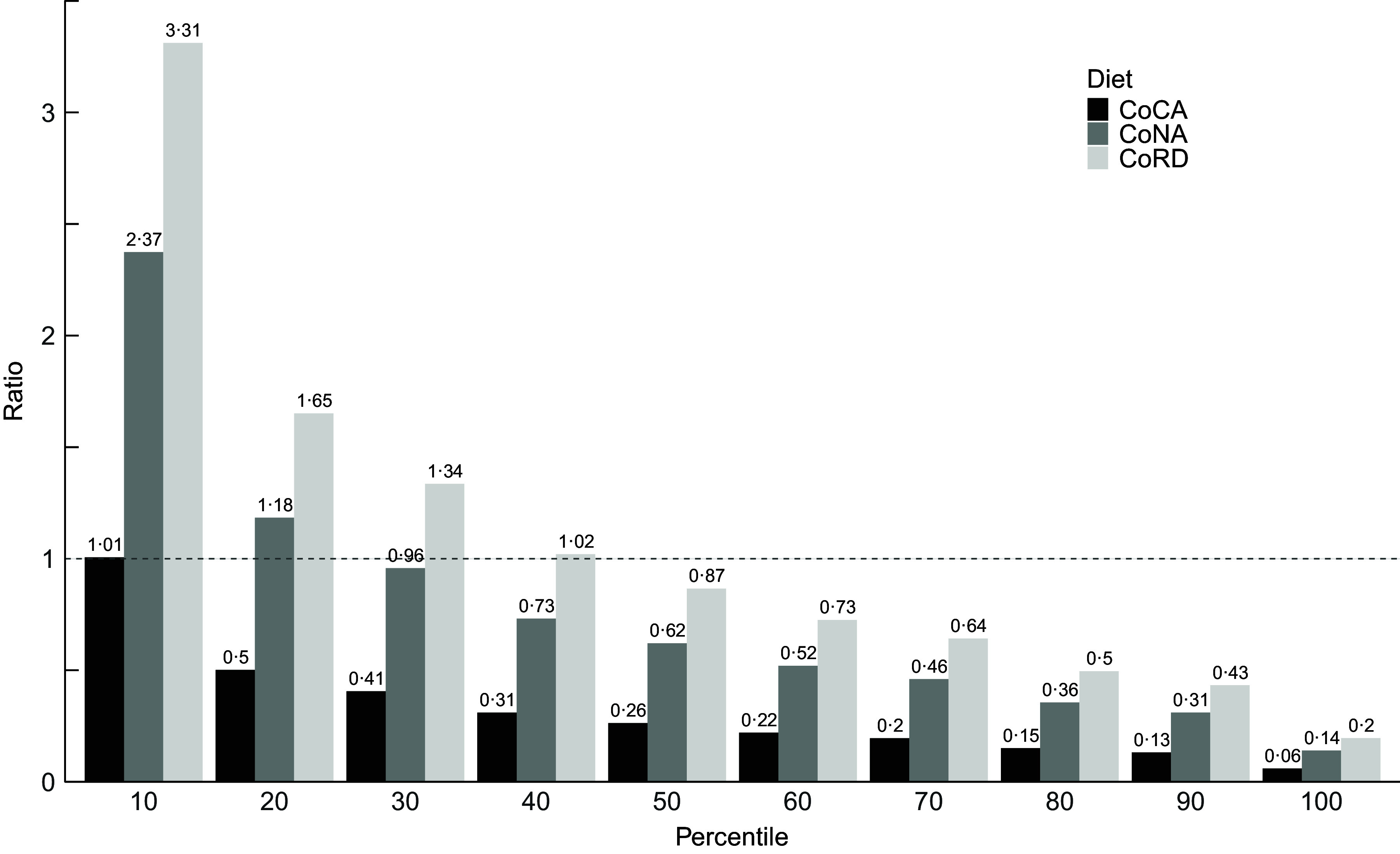
Ratio of the per capita cost to the average per capita expenditure on food. The ratio of the estimated per capita minimum cost (USD) of a diet for households to the average per capita expenditure on food (USD) by households at each income level. Source: Own calculations based on the information of the study.

Figure 5 shows the indicator of the ratio of the estimated per capita minimum cost of a diet for households to the average per capita expenditure on food by households at each income level. The per capita CoCA is 1·01 times the average per capita food expenditure for households in the 10th percentile, 26 % of spending for households in the 50th percentile and approximately 15 % for those in the 80th percentile. This observation verifies the limitations of households in the 10th percentile in terms of their ability to afford a subsistence diet. The CoNA is more than twice the average per capita food expenditure for households in the 10th percentile and 1·18 times for households in the 20th percentile. Similarly, there is a lack of affordability for households between the 10th and 40th percentiles of the CoRD. For households in the 50th percentile, the affordability of a healthy diet is likely to be constrained, as the per capita CoRD represents approximately 87 % of their average per capita food expenditure.

Affordability outcomes are sensitive to the share of household food spending. To provide a conservative lower-bound estimate, we assume urban households allocate their entire income to food. Under this assumption, only households in the lowest income group (10th percentile) face challenges in affording the least-cost diets: 13·71 % cannot afford the CoCA; 42·22 % the CoNA and over 67 % a healthy diet. The cost-to-expense ratio shows that the per capita CoNA accounts for 93 % of the average per capita food expenditure for this group. Therefore, even if all income were allocated to food, the per capita CoRD would still exceed the average per capita food expenditure by 1·29 times.

## Discussion

This study determines the cost and affordability of three levels of diet quality for urban households in Cali, Colombia, using least-cost diet metrics. We identified significant cost variations across demographic groups categorised by age, sex and physiological condition. Adhering to Colombia’s FBDG recommendations requires a premium to meet energy and nutrient requirements. Only 63·92 % of urban households in Cali can afford a healthy diet, revealing severe economic barriers to affordable nutrition for over 30 % of the population. The cost-to-expense ratio further demonstrates that reallocating expenditures towards diet is insufficient to close the affordability gap for lower-income households. These key findings are discussed in detail below.

First, our study reveals notable variations in least-cost diet metrics across demographic groups. Consistent with previous research^(3,9)^, the CoCA metric is calculated by multiplying the price per kilocalorie of the least-cost starchy staple – in this case, a specific variety of rice – by the individual’s EER. As expected, the daily CoCA is notably higher for men in the 19–30 group due to their increased energy needs. Unlike studies that estimate the CoNA based on a representative unit – e.g. agent^(2,18,35)^ or household^(14,15,21,22,24)^ – our analysis accounts for the influence of demographic and physiological factors on dietary requirements, revealing significantly a higher daily CoNA for adolescents and lactating or pregnant women than for other consumers. These barriers to access, particularly for the latter groups, constitute a substantial public health concern, as they may increase the risk of adverse pregnancy outcomes and contribute to developmental delays and cognitive impairments in children^(38)^.

To account for variations in daily CoNA due to differences in EER across demographic groups, we also express the CoNA on a per 1000 kcal basis. Our results, which are in line with a previous recent study^(20)^, reveal an N-shaped trajectory in CoNA per 1000 kcal and that CoNA per 1000 kcal, for individuals aged 19 years and older, is generally higher for women than for men, indicating that a diet with a higher density of nutrients with locally available foods tends to be more costly for women.

**Figure 6.**
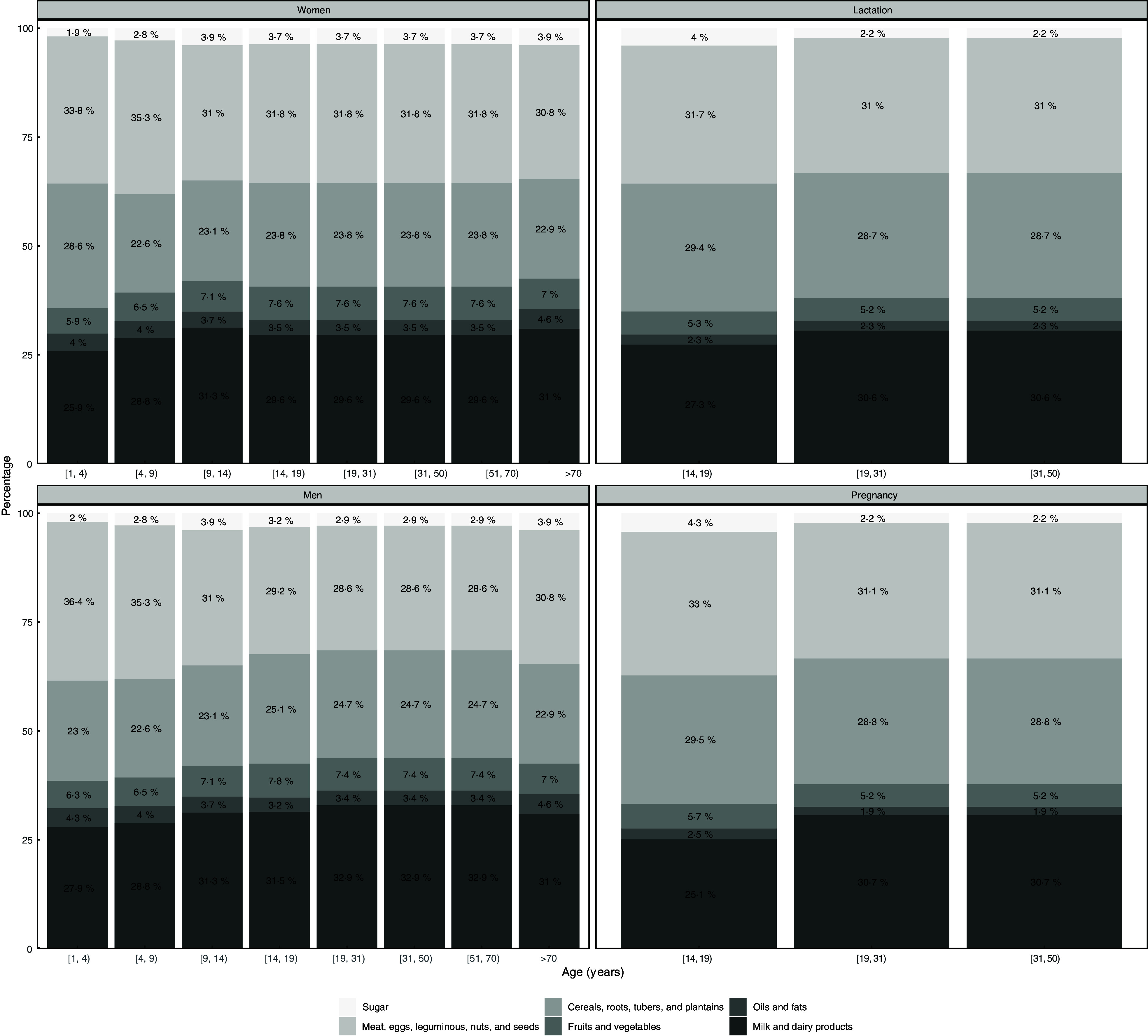
Share (%) of total cost for each food group in the recommended diet, across demographic groups by age, sex and physiological condition. Source: Own calculations based on the information of the study.

Our study indicates that a healthy diet ensures nutrient adequacy with some variability, meeting about 83·2 % of the lower limits of nutrient requirements (±10 %) (see online supplementary material, Supplemental Table S6 for CoRD validation). This finding corresponds to prior research showing that such diets typically fulfill over 80 % of nutrient requirements on average^(39)^, and it is further corroborated by a global study reporting 89 % (±5 %) fulfillment of nutrient needs^(17)^. Notably, adolescent girls aged 14–18 years and adult men of 19–30 years have to deal with a higher daily CoRD, driven by the cost of meeting dietary recommendations for two specific food groups: (i) meat, eggs, legumes, nuts and seeds and (ii) milk and dairy products. These two groups, along with the fruits and vegetables group, represent around 70 % of the total cost of a healthy diet (Figure 6), corroborating prior research that identifies these food groups as major contributors to the CoRD^(18,39)^.

Second, our findings reveal the significant additional costs that urban households in Cali may incur in their efforts to improve diet quality. Specifically, the CoRD was on average 1·4 (sd: 0·18) times higher than the CoNA, indicating an additional expense associated with aligning dietary intake with the recommendations outlined in the Colombian FBDG (Table 5). This concurs with a recent study on South American countries that, using a representative agent approach, reported an identical CoRD:CoNA ratio of 1·4^(40)^. Furthermore, our analysis revealed that CoNA exceeded CoCA by a factor of 2·4 (sd: 0·26), indicating a pronounced nutrient premium. This premium surpasses the CoNA:CoCA ratios documented in upper-middle income countries (2·18 (sd: 0·48))^(17)^ and in the Latin American and Caribbean region (2·21 (sd: 0·73))^(3)^. These results underscore the economic constraints and context-specific challenges that may hinder dietary quality improvements, nutritional adequacy achievement and the adoption of healthy dietary patterns among urban households in Cali.

**Table 5.** Ratios of nutrient-adequate diet cost to caloric-adequate diet cost (CoNA/CoCA), recommended diet cost to caloric-adequate diet cost (CoRD/CoCA), and recommended diet cost to nutrient-adequate diet cost (CoRD/CoNA) across demographic groups by age, sex, and physiological condition

	Women			Pregnancy			Lactating			Men		
Group	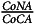	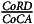	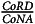	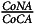	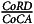	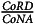	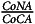	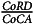	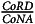	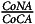	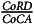	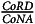
(1–4)	1·95	3·62	1·85	–	–	–	–	–	–	2·69	3·36	1·25
(4–9)	2·74	3·33	1·22	–	–	–	–	–	–	2·58	3·33	1·29
(9–14)	2·66	3·26	1·23	–	–	–	–	–	–	2·66	3·26	1·23
(14–19)	2·16	3·26	1·51	2·61	3·15	1·20	2·33	3·20	1·37	2·21	3·30	1·49
(19–31)	2·36	3·26	1·38	2·28	3·41	1·50	2·10	3·38	1·61	2·04	3·34	1·64
(31–51)	2·45	3·26	1·33	2·38	3·41	1·43	2·15	3·38	1·57	2·07	3·34	1·61
(51–70)	2·75	3·26	1·19	–	–	–	–	–	–	2·40	3·34	1·39
≥ 70	2·82	3·23	1·15	–	–	–	–	–	–	2·40	3·23	1·34

Source: Own calculations based on the information of the study.

Third, our analysis identifies vitamin C, vitamin A, vitamin B_12_ and Ca as the key nutrients limiting the daily CoNA across demographic groups. While this is consistent with studies in other countries^(12,41)^, variations in local food availability, retail prices and seasonal factors have led other research to identify different limiting nutrients, such as riboflavin^(25)^; Fe in children^(35)^; and biotin, molybdenum, potassium, selenium and pantothenic acid^(41)^. Limiting nutrients often correlates with potential nutritional deficiencies, providing valuable insights for interventions to enhance access to micronutrient-rich foods. Long-term strategies should promote dietary diversity by supporting local production and consumption of varied, nutrient-rich foods. Policy measures, such as fiscal subsidies targeted at producers of foods that are under-consumed relative to recommended dietary levels for a healthy diet (i.e. priority foods), can enhance availability and encourage consumption^(1)^. Additionally, medium- and short-term measures may include the fortification of mass-consumed staple foods, home fortification, biofortification and supplementation for vulnerable groups^(42)^. Colombia has implemented food fortification strategies including fortifying wheat flour with thiamine, niacin, riboflavin, folate and Fe under Decree 1944 of 1966. Social programs also have included fortifying specific foods, such as vegetables mixes with added micronutrients, and milk and cookies with folic acid, Fe and Zn.

Fourth, our study underscores the limitations of conventional poverty measures based solely on energy standards, revealing that least-cost diet metrics offer a more comprehensive assessment of the challenges households face in achieving adequate nutrition and a healthy diet. National poverty lines are typically established by estimating the expenditure required for a consumption bundle deemed adequate for basic food and non-food needs^(43)^. The food component establishes the extreme poverty line, based on a basket defined by the reference population’s consumption patterns and adjusted to meet the per capita daily caloric requirement. During the study period, the extreme poverty line in Cali was estimated at 58·36 USD (220 911·40 COP)^(44)^. In comparison, the monthly per capita CoNA was 49·84 USD (188 649·10 COP), exceeding 80 % of this threshold, while the CoRD was 69·51 USD (261 114·50 COP), surpassing it by 1·2 times. These findings reveal that not only poor households but also near-poor households – classified as non-poor under conventional poverty lines – cannot afford at least a least-cost healthy diet, as confirmed by previous studies^(17–19,45)^. This has significant implications for nutrition and food security policy aimed at reducing hunger and malnutrition, as conventional poverty lines may misguide program targets by failing to capture the broader economic constraints affecting dietary quality.

Fifth, our analysis highlights both the challenges households face in affording the three increasing levels of diet quality and the difficulty in closing the affordability gap. Households in the first two quintiles have average per capita food expenditures that fall below both the monthly per capita CoNA and CoRD. This issue is especially severe for households in the 10th percentile, where even by allocating 100 % of their income to food, the per capita CoRD exceeds their average per capita food expenditure by 1·29 times (see online supplementary material, Supplemental Table S7). Therefore, closing the affordability gap cannot be achieved merely by reallocating food expenditures, i.e. without an increase in the current household income level.

Our methodology offers several key strengths in analysing affordability. First, unlike global studies that rely on country-level data^(3,18)^, we use local food availability, retail prices, nutritional recommendations, income distribution and household food expenditure, contributing to increased relevance and accuracy in our analysis. Second, our focus on the representative household, as opposed to a single representative agent, allows our affordability indicators to be sensitive to variations in household size, providing a deeper understanding of diet costs. Additionally, in line with prior research^(15,21,22)^, we employ individualised diets for each household member, which contrasts with the shared diet approach proposed in recent literature^(23)^. This distinction means that our estimates offer a lower bound of household diet costs, whereas the shared diet method, in accordance with Rawls’ maximin principle, would produce upper-bound estimates by focusing on the nutritional needs of the neediest household member. This approach would likely lead to less favourable affordability outcomes, particularly for low- and middle-income economies.

Our approach has limitations. First, the locally available food set, defined from a supply-side perspective, would benefit from integrating demand-side data^(46,47)^ on commonly consumed foods and quantities. Incorporating population consumption patterns through model constraints could better align least-cost diets with cultural dietary habits^(15,22)^. However, the assumption that households will maintain unchanged consumption patterns while improving diet quality is problematic. For instance, evidence from Colombia indicates a significant misalignment between current dietary patterns and healthy diet recommendations, particularly among lower-income households, whose eating patterns include a higher proportion of energy-dense foods^(48)^. Adopting a healthier diet would require shifts in consumption patterns that, though influenced by multiple factors, remain largely constrained by income. Second, assuming constant retail mark-ups within food categories for estimating retail prices, due to a lack of publicly available data in Colombia, does not fully capture retail pricing complexities, potentially introducing deviations in the affordability indicators based on the estimated least-cost diet metrics. Although useful under stable market conditions – where mark-ups are likely similar due to factors like shelf life, spoilage risks and supply stability – this assumption may overlook variations from demand elasticity and market dynamics. Previous research^(49,50)^ also highlights the challenges with using national averages or prevailing retail prices for diet cost estimation. Future research should focus on dynamic pricing models to enhance accuracy. Third, the distribution of monthly per capita household income in our study, though closely aligned with Great Integrated Household Survey data used by the National Administrative Department of Statistics for poverty incidence rates, lacks supplementary administrative records. For September 2022, our estimates indicate 4·66 % of households in Cali in extreme poverty and 18·65 % in monetary poverty, compared to 5·6 % and 19·80 % reported by official statistics. Minor discrepancies may arise from the absence of additional data on pension payments and institutional aid programs, including conditional cash transfers. Similar minor discrepancies might be expected in affordability indicators.

This study highlights the significant barriers urban households in Cali, Colombia face in affording three increasing levels of diet quality. Our analysis reveals notable variations in least-cost diet metrics across demographic groups, with additional costs required to meet nutrient requirements and adhere to national FBDG recommendations. Using a representative household approach with individualised diets, this study demonstrates the substantial difficulties faced by lower-income households in affording caloric-adequate, nutrient-adequate and recommended diets, even with food expenditure reallocation. This approach provides key insights into local food system issues and informs the design and implementation of targeted public policies for the case study. Given the scarcity of similar studies in Latin America, this research establishes a valuable methodological basis for conducting analogous investigations in the region, while expanding knowledge on the food and nutritional security challenges faced by urban households in Cali.

## Supporting information

Yoshioka Vargas et al. supplementary material Yoshioka Vargas et al. supplementary material

## Supplementary material

For supplementary material accompanying this paper visit https://doi.org/10.1017/S1368980025000564

## References

[1] FAO, IFAD, UNICEF et al. (2022) The State of Food Security and Nutrition in the World. Repurposing Food and Agricultural Policies to Make Healthy Diets More Affordable. Rome: FAO.

[2] Hirvonen K , Bai Y , Headey D et al. (2020) Affordability of the EAT–Lancet reference diet: a global analysis. Lancet Glob Health 8, e59–66. 31708415 10.1016/S2214-109X(19)30447-4 PMC7024996

[3] Bai Y , Alemu R , Block SA et al. (2021) Cost and affordability of nutritious diets at retail prices: evidence from 177 countries. Food Policy 99, 101983. 33767525 10.1016/j.foodpol.2020.101983 PMC7970354

[4] Lasdun V , Harou AP & Magomba C (2023) COVID-19, climate shocks, and food security linkages: evidence and perceptions from smallholder farming communities in Tanzania. Environ Dev Econ 28, 211–229.

[5] Lin F , Li X , Jia N et al. (2023) The impact of Russia-Ukraine conflict on global food security. Glob Food Sec 36, 100661.

[6] Vos R , Glauber J , Hernández M et al. (2022) COVID-19 and food inflation scares. In Global Food Security: Two Years Later, pp. 64–72 [ J McDermott and J Swinnen , editors]. Washington, DC: International Food Policy Research Institute.

[7] Aksoy MA & Isik-Dikmelik A (2008) *Are Low Food Prices Pro-Poor? Net Food Buyers and Sellers in Low-Income Countries*. Policy Research Working Paper, Report No.: 4642. Washington, DC: World Bank.

[8] Ivanic M & Martin W (2008) Implications of higher global food prices for poverty in low-income countries. Agric Econ 39, 1405–1416.

[9] Mahrt K , Mather D , Herforth A et al. (2019) *Household Dietary Patterns and the Cost of a Nutritious Diet in Myanmar*. IFPRI Discussion Paper, Report No.: 01854. Washington, DC: IFPRI.

[10] Kachwaha S , Nguyen PH , DeFreese M et al. (2020) Assessing the economic feasibility of assuring nutritionally adequate diets for vulnerable populations in Uttar Pradesh, India: findings from a ‘cost of the diet’ analysis. Curr Dev Nutr 4, 1–9. 33313474 10.1093/cdn/nzaa169 PMC7721462

[11] Deptford A , Allieri T , Childs R et al. (2017) Cost of the Diet: a method and software to calculate the lowest cost of meeting recommended intakes of energy and nutrients from local foods. BMC Nutr 3, 1–17. 10.1186/s40795-017-0136-4 PMC7050783 32153808

[12] Masters WA , Bai Y , Herforth A et al. (2018) Measuring the affordability of nutritious diets in Africa: price indexes for diet diversity and the cost of nutrient adequacy. Am J Agric Econ 100, 1285–1301. 32139915 10.1093/ajae/aay059 PMC7053386

[13] Chungchunlam SMS , Moughan PJ , Garrick DP et al. (2020) Animal-sourced foods are required for minimum-cost nutritionally adequate food patterns for the United States. Nat Food 1, 376–381. 37128091 10.1038/s43016-020-0096-8

[14] de Pee S , Hardinsyah R , Jalal F et al. (2021) Balancing a sustained pursuit of nutrition, health, affordability, and climate goals: exploring the case of Indonesia. Am J Clin Nutr 114, 1686–1697. 34477830 10.1093/ajcn/nqab258 PMC8574631

[15] Biehl E , Klemm RDW , Manohar S et al. (2016) What does it cost to improve household diets in Nepal? Using the cost of the diet method to model lowest cost dietary changes. Food Nutr Bull 37, 247–260. 27378799 10.1177/0379572116657267

[16] Dizon F , Herforth A & Wang Z (2019) The cost of a nutritious diet in Afghanistan, Bangladesh, Pakistan, and Sri Lanka. Glob Food Sec 21, 38–51.

[17] Herforth A , Venkat A , Bai Y et al. (2022) *Methods and Options to Monitor the Cost and Affordability of a Healthy Diet Globally. Background Paper for the State of Food Security and Nutrition in the World 2022*. FAO Agricultural Development Economics Working Paper No.: 22–03. Rome: FAO.

[18] Herforth A , Bai Y , Venkat A et al. (2020) *Cost and Affordability of Healthy Diets Across and within Countries. Background Paper for the State of Food Security and Nutrition in the World 2020*. FAO Agricultural Development Economics Technical Study, Report No.: 9. Rome: FAO.

[19] FAO, IFAD, UNICEF et al. (2020) The State of Food Security and Nutrition in the World. Transforming Food Systems for Affordable Healthy Diets. Rome: FAO.

[20] Bai Y , Herforth A & Masters WA (2022) Global variation in the cost of a nutrient-adequate diet by population group: an observational study. Lancet Planet Health 6, e19–e28. 34998455 10.1016/S2542-5196(21)00285-0 PMC8753783

[21] Geniez P , Mathiassen A , De Pee S et al. (2014) Integrating food poverty and minimum cost diet methods into a single framework: a case study using a Nepalese household expenditure survey. Food Nutr Bull 35, 151–159. 25076762 10.1177/156482651403500201

[22] Giacobone G , Tiscornia MV , Guarnieri L et al. (2021) Measuring cost and affordability of current *v.* healthy diets in Argentina: an application of linear programming and the INFORMAS protocol. BMC Public Health 21, 1–9. 33971851 10.1186/s12889-021-10914-6 PMC8111730

[23] Schneider KR , Christiaensen L , Webb P et al. (2021) *Assessing the Affordability of Nutrient-Adequate Diets*. Policy Research Working Paper, Report No.: 9834. Washington, DC: World Bank Group.

[24] Baldi G , Martini E , Catharina M et al. (2013) Cost of the Diet (CoD) tool: first results from Indonesia and applications for policy discussion on food and nutrition security. Food Nutr Bull 34, S35–S42. 24049994 10.1177/15648265130342S105

[25] Frega R , Lanfranco JG , De Greve S et al. (2012) What linear programming contributes: world food programme experience with the ‘cost of the diet’ tool. Food Nutr Bull 33, S228–S234. 23193775 10.1177/15648265120333S212

[26] Rankin S , Hurtado LJ , Bonilla-Findji O et al. (2021) *Perfil del Sistema Alimentario de Cali, ciudad-región (Food System Profile of the Cali City-Region)*. Alliance of Bioversity International and CIAT Report No. 27. Cali, Colombia: Alliance of Bioversity International and CIAT.

[27] Departamento Administrativo Nacional de Estadística (DANE) (2022) Sistema de Información de precios y abastecimiento del sector agropecuario (Price and supply information system of the agricultural and livestock sector). https://www.dane.gov.co/index.php/estadisticas-por-tema/agropecuario/sistema-de-informacion-de-precios-sipsa (accessed July 2023).

[28] Instituto Colombiano de Bienestar Familiar (ICBF) (2018) Tabla de Composición de Alimentos Colombianos (TCAC). https://www.icbf.gov.co/system/files/tcac_web.pdf (accessed July 2023).

[29] FAO/INFOODS (2012) Guidelines for Food Matching. Version 1.2. Rome: FAO.

[30] Instituto Colombiano de Bienestar Familiar, Ministerio de Salud y Protección Social, Departamento Administrativo para la Prosperidad Social et al. (2015) Encuesta Nacional de Situación Nutricional - ENSIN (National Survey of Nutritional Status). https://www.icbf.gov.co/bienestar/nutricion/encuesta-nacional-situacion-nutricional#ensin3 (accessed July 2023).

[31] Ministerio de Salud y Protección Social (2015) Estudio Nacional de Salud, Bienestar y Envejecimiento (National Study of Health, Well-Being and Aging). https://www.minsalud.gov.co/sites/rid/lists/bibliotecaDigital/RIDE/VS/ED/GCFI/Resumen-ejecutivo-encuesta-SABE.pdf (accessed July 2023).

[32] Ministerio de Salud y Protección Social (2016) Resolución 3803 de 2016 por la cual se establecen las Recomendaciones de Ingesta de Energía y Nutrientes- RIEN (Resolution 3803 of 2016: Recommendations for Energy and Nutrient Intake - RIEN). https://www.minsalud.gov.co/Normatividad_Nuevo/Resoluci%C3%B3n%203803%20de%202016.pdf (accessed July 2023).

[33] Institute of Medicine (IOM) (2006) Dietary Reference Intakes: The Essential Guide to Nutrient Requirements. Washington, DC: The National Academies Press.

[34] Paris Q (2016) The Diet Problem Revisited. An Economic Interpretation of Linear Programming, pp. 241–253. New York: Palgrave Macmillan US.

[35] Briend A , Darmon N , Ferguson E et al. (2003) Linear programming: a mathematical tool for analyzing and optimizing children’s diets during the complementary feeding period. J Pediatr Gastroenterol Nutr 36, 12–22. 12499991 10.1097/00005176-200301000-00006

[36] ICBF & FAO (2020) Guías Alimentarias Basadas en Alimentos para la población colombiana mayor de 2 años (Colombian Food-Based Dietary Guidelines). https://www.icbf.gov.co/guias-alimentarias-basadas-en-alimentos-para-la-poblacion-colombiana-mayor-de-2-anos (accessed July 2023).

[37] Departamento Administrativo Nacional de Estadística (DANE) (2022) Gran Encuesta Integrada de Hogares (Great Integrated Household Survey). https://microdatos.dane.gov.co/index.php/catalog/771 (accessed July 2023).

[38] Gebre B , Biadgilign S , Taddese Z et al. (2018) Determinants of malnutrition among pregnant and lactating women under humanitarian setting in Ethiopia. BMC Nutr 4, 1–8. 32153875 10.1186/s40795-018-0222-2 PMC7050776

[39] Van DTT , Herforth AW , Trinh HT et al. (2024) Cost and affordability of healthy diets in Vietnam. Public Health Nutr 27, e3. 10.1017/S1368980023002665 PMC10830355 38037710

[40] FAO, IFAD, UNICEF et al. (2021) The State of Food Security and Nutrition in the World. Transforming Food Systems for Food Security, Improved Nutrition and Affordable Healthy Diets for All. Rome: FAO.

[41] Chungchunlam SMS , Garrick DP & Moughan PJ (2021) Using linear programming to determine the role of plant and animal-sourced foods in least-cost, nutritionally adequate diets for adults. Curr Dev Nutr 5, nzab132. 34870073 10.1093/cdn/nzab132 PMC8634088

[42] Ministerio de la Salud y Protección Social de Colombia (2015) Estrategia nacional para la prevención y control de las deficiencias de micronutrientes en Colombia 2014–2021 (National Strategy for the Prevention and Control of Micronutrient Deficiencies in Colombia 2014–2021). Bogotá, DC: Ministerio de la Salud y Protección Social de Colombia.

[43] Ravallion M (2010) *Poverty Lines across the World*. Policy Research Working Paper, Report No.: 5284. Washington, DC: World Bank.

[44] Departamento Administrativo Nacional de Estadística (DANE) (2022) Pobreza monetaria y pobreza monetaria extrema (Monetary Poverty and Extreme Poverty). https://www.dane.gov.co/index.php/estadisticas-por-tema/pobreza-y-condiciones-de-vida/pobreza-monetaria (accessed July 2023).

[45] Mahrt K , Herforth AW , Robinson S et al. (2022) *Nutrition as a Basic Need. A New Method for Utility-Consistent and Nutritionally Adequate Food Poverty Lines*. IFPRI Discussion Paper, Report No.: 02120. Washington, DC: IFPRI.

[46] Starck CS , Blumfield M , Keighley T et al. (2021) Nutrient dense, low-cost foods can improve the affordability and quality of the New Zealand diet—a substitution modelling study. Int J Environ Res Public Health 18, 7950. 34360243 10.3390/ijerph18157950 PMC8345759

[47] Lauk J , Nurk E , Robertson A et al. (2020) Culturally optimised nutritionally adequate food baskets for dietary guidelines for minimum wage Estonian families. Nutrients 12, 2613. 32867197 10.3390/nu12092613 PMC7551125

[48] Mora-García G , Ruiz-Díaz MS , Villegas R et al. (2020) Changes in diet quality over 10 years of nutrition transition in Colombia: analysis of the 2005 and 2015 nationally representative cross-sectional surveys. Int J Public Health 65, 547–558. 32632458 10.1007/s00038-020-01396-1

[49] Darmon N & Drewnowski A (2015) Contribution of food prices and diet cost to socioeconomic disparities in diet quality and health: a systematic review and analysis. Nutr Rev 73, 643–660. 26307238 10.1093/nutrit/nuv027 PMC4586446

[50] Timmins KA , Morris MA , Hulme C et al. (2013) Comparability of methods assigning monetary costs to diets: derivation from household till receipts *v.* cost database estimation using 4-day food diaries. Eur J Clin Nutr 67, 1072–1076. 24022262 10.1038/ejcn.2013.157

