# Supplementary material for: Cost and affordability of three levels of diet quality for urban households in Colombia
Source: Public Health Nutr. 2025 Jun 2;29(1):e70. doi: 10.1017/S1368980025000564 (PMC13289846; doi:10.1017/S1368980025000564)
Supplement: Yoshioka Vargas et al. supplementary material [file S1368980025000564sup001.docx]

**Supplementary material: Cost and affordability of three levels of diet quality for urban households in Colombia**

**Table S1.** Locally-available food prices and nutrient composition

| **Food** | **Group** | **Serving (g)** | **Price** | **Energy (Kcal)** | **Protein (g)** | **CHO (g)** | **Fats(g)** | **Calcium (mg)** | **Zinc (mg) (mg)** | **Iron (mg)** | **Magnesium (mg)** | **Phosphorus (mg)** | **Vitamin C (mg)** | **Thiamine (mg)** | **Riboflavin (mg)** | **Niacin (mg)** | **Folate (mcg)** | **Vitamin B12 (mcg)** | **Vitamin A (EAR)** | **Sodium (mg)** |
| --- | --- | --- | --- | --- | --- | --- | --- | --- | --- | --- | --- | --- | --- | --- | --- | --- | --- | --- | --- | --- |
| Vegetable oil blend | D | 100 | 0.35 | 900 | 0 | 0 | 100 | 0 | 0 | 0 | 0 | 1 | 0 | 0 | 0 | 0 | 0 | 0 | 0 | 0 |
| Avocado | C | 100 | 0.23 | 179 | 1.6 | 10 | 13.3 | 10 | 0.5 | 0.4 | 27 | 40 | 8 | 0.05 | 0.12 | 1.4 | 81 | 0 | 3 | 6 |
| Pumpkin | B | 100 | 0.06 | 30 | 0.8 | 5.8 | 0.2 | 20 | 0.2 | 0.8 | 12 | 34 | 9 | 0.05 | 0.05 | 0.6 | 16 | 0 | 1775 | 1 |
| Celery | B | 100 | 0.11 | 26 | 0.7 | 4.6 | 0.2 | 42 | 0.2 | 0.4 | 14 | 33 | 5 | 0.04 | 0.04 | 0.3 | 36 | 0 | 22 | 80 |
| Yellow Arracacha | B | 100 | 0.20 | 111 | 0.9 | 26.3 | 0.1 | 21 | 0 | 0.9 | 12 | 52 | 20 | 0.06 | 0.06 | 3.8 | 15.6 | 0 | 57 | 1 |
| Rice | A | 100 | 0.11 | 353 | 6.7 | 80.1 | 0.4 | 9 | 1.2 | 0.8 | 35 | 140 | 0 | 0.07 | 0.03 | 1.8 | 9 | 0 | 0 | 2 |
| Green peas | B | 100 | 0.66 | 99 | 5.9 | 15.5 | 0.3 | 36 | 1.2 | 1.5 | 33 | 110 | 33 | 0.36 | 0.12 | 2.2 | 65 | 0 | 51 | 5 |
| Imported dry green peas | T | 100 | 0.14 | 374 | 23.9 | 60.2 | 1.1 | 60 | 3.5 | 4.6 | 119 | 346 | 2 | 0.78 | 0.16 | 3.1 | 274 | 0 | 22 | 15 |
| Oatmeal flakes | A | 100 | 0.31 | 411 | 16.9 | 64.1 | 7.5 | 54 | 4 | 4.5 | 155 | 320 | 0 | 0.72 | 0.13 | 1.6 | 57 | 0 | 0 | 3 |
| Ground oats | A | 100 | 0.31 | 414 | 14.7 | 66.2 | 8.6 | 54 | 3.2 | 4 | 132 | 440 | 0 | 0.63 | 0.13 | 1.5 | 32 | 0 | 0 | 19 |
| Brown sugar | K | 100 | 0.13 | 390 | 0 | 97.5 | 0 | 85 | 0.2 | 1.9 | 24 | 22 | 0 | 0.01 | 0.01 | 0.1 | 1 | 0 | 0 | 39 |
| Sugar | K | 100 | 0.12 | 397 | 0 | 99.3 | 0 | 0 | 0 | 0.1 | 1 | 0 | 0 | 0 | 0 | 0 | 0 | 0 | 0 | 0 |
| Banana | C | 100 | 0.07 | 101 | 1.5 | 22.3 | 0.1 | 8 | 0.2 | 0.9 | 29 | 27 | 19 | 0.05 | 0.03 | 0.6 | 21 | 0 | 22 | 3 |
| Basa. imported frozen fillet | E | 100 | 0.45 | 85 | 18.3 | 0.1 | 1.3 | 27 | 0.3 | 0.8 | 24 | 186 | 0 | 0.06 | 0.07 | 1.3 | 12 | 1.2 | 17 | 75 |
| Broccoli | B | 100 | 0.36 | 46 | 3 | 6.6 | 0.3 | 60 | 0.4 | 1.1 | 21 | 70 | 89 | 0.07 | 0.14 | 0.9 | 63 | 0 | 31 | 33 |
| Instant coffee | L | 100 | 4.55 | 355 | 13 | 74.7 | 0.4 | 141 | 0.4 | 4.4 | 326 | 303 | 0 | 0.01 | 0.08 | 28.2 | 1 | 0 | 0 | 38 |
| Ground coffee | L | 100 | 0.80 | 479 | 14.2 | 67.5 | 12.3 | 130 | 0.5 | 5.8 | 165 | 223 | 0 | 0.07 | 0.1 | 31.6 | 3 | 0 | 0 | 1 |
| Boneless pork. leg | F | 100 | 0.47 | 115 | 20.3 | 0 | 3.8 | 10 | 2.4 | 0.5 | 20 | 203 | 0 | 0.92 | 0.29 | 6.9 | 3 | 0.61 | 2 | 87 |
| Pork. back ribs | F | 100 | 0.57 | 277 | 18.7 | 0 | 22.5 | 11 | 1.8 | 1 | 20 | 180 | 0 | 0.62 | 0.26 | 5.1 | 4 | 0.87 | 5 | 93 |
| Pork. spine | F | 100 | 0.33 | 277 | 18.7 | 0 | 22.5 | 11 | 1.8 | 1 | 20 | 180 | 0 | 0.62 | 0.26 | 5.1 | 4 | 0.87 | 5 | 93 |
| Boneless pork. loin | F | 100 | 0.51 | 110 | 21.6 | 0.3 | 2.5 | 10 | 1.9 | 0.9 | 27 | 216 | 0 | 0.95 | 0.34 | 7.1 | 0 | 0.51 | 0 | 53 |
| Boneless pork. shank | F | 100 | 0.49 | 131 | 20.5 | 0.2 | 5.4 | 8 | 2.5 | 1.2 | 23 | 217 | 0 | 0.8 | 0.24 | 5.7 | 5 | 1 | 0 | 57 |
| Pork. bacon jowl | F | 100 | 0.33 | 784 | 2.9 | 0 | 85.8 | 6 | 0.8 | 1.1 | 4 | 37 | 0 | 0.23 | 0.17 | 2.7 | 1 | 0.18 | 10 | 22 |
| Beef. shoulder | F | 100 | 0.73 | 121 | 21.8 | 0 | 3.8 | 20 | 6.9 | 2.3 | 14 | 204 | 0 | 0.1 | 0.22 | 5.7 | 8 | 3.33 | 0 | 63 |
| Beef. round | F | 100 | 0.73 | 121 | 21.8 | 0 | 3.8 | 20 | 6.9 | 2.3 | 14 | 204 | 0 | 0.1 | 0.22 | 5.7 | 8 | 3.33 | 0 | 63 |
| Beef. sirloin | F | 100 | 0.73 | 223 | 21.6 | 0.3 | 15.1 | 6 | 3.5 | 1.9 | 13 | 182 | 0 | 0.06 | 0.17 | 4.3 | 3 | 2.13 | 2 | 50 |
| Beef. strip loin | F | 100 | 0.96 | 139 | 21.8 | 0 | 5.7 | 6 | 3.6 | 2.7 | 18 | 215 | 0 | 0.08 | 0.23 | 5.1 | 7 | 2.65 | 0 | 51 |
| Beef. ribs | F | 100 | 0.63 | 287 | 17.1 | 2.3 | 23.3 | 9 | 3.5 | 1.6 | 16 | 176 | 0 | 0.1 | 0.18 | 4.1 | 5 | 2.34 | 5 | 60 |
| Beef. flank | F | 100 | 0.47 | 223 | 21.6 | 0.3 | 15.1 | 6 | 3.5 | 1.9 | 13 | 182 | 0 | 0.06 | 0.17 | 4.3 | 3 | 2.13 | 2 | 50 |
| Beef. stickin | F | 100 | 0.67 | 257 | 18.4 | 0.2 | 20.3 | 8 | 3.5 | 1.5 | 19 | 174 | 0 | 0.09 | 0.16 | 3.2 | 6 | 2.22 | 0 | 58 |
| Beef. chuck | F | 100 | 0.68 | 121 | 21.8 | 0 | 3.8 | 20 | 6.9 | 2.3 | 14 | 204 | 0 | 0.1 | 0.22 | 5.7 | 8 | 3.33 | 0 | 63 |
| Beef. brisket | F | 100 | 0.65 | 223 | 21.6 | 0.3 | 15.1 | 6 | 3.5 | 1.9 | 13 | 182 | 0 | 0.06 | 0.17 | 4.3 | 3 | 2.13 | 2 | 50 |
| Beef. rump roast | F | 100 | 0.94 | 223 | 21.6 | 0.3 | 15.1 | 6 | 3.5 | 1.9 | 13 | 182 | 0 | 0.06 | 0.17 | 4.3 | 3 | 2.13 | 2 | 50 |
| Beef. flank steak | F | 100 | 0.65 | 257 | 18.4 | 0.2 | 20.3 | 8 | 3.5 | 1.5 | 19 | 174 | 0 | 0.09 | 0.16 | 3.2 | 6 | 2.22 | 0 | 58 |
| Scallion (*Aquitania*) | B | 100 | 0.18 | 37 | 1.2 | 6.8 | 0.1 | 57 | 0.2 | 0.5 | 17 | 31 | 13 | 0.04 | 0.05 | 0.3 | 26 | 0 | 200 | 15 |
| Scallion (*Tenerife)* | B | 100 | 0.21 | 37 | 1.2 | 6.8 | 0.1 | 57 | 0.2 | 0.5 | 17 | 31 | 13 | 0.04 | 0.05 | 0.3 | 26 | 0 | 200 | 15 |
| Bitter chocolate | L | 100 | 0.78 | 622 | 4.9 | 49.3 | 42.6 | 80 | 2.9 | 3.6 | 186 | 220 | 0 | 0.04 | 0.04 | 1.1 | 6 | 0.28 | 2 | 17 |
| Cob | A | 100 | 0.11 | 152 | 3.4 | 30.5 | 1.2 | 5 | 0.4 | 0.8 | 34 | 120 | 10 | 0.13 | 0.07 | 1.6 | 42 | 0 | 28 | 3 |
| Coconut | C | 100 | 0.19 | 402 | 3.6 | 12.6 | 35.5 | 7 | 0.8 | 1.3 | 40 | 80 | 5 | 0.05 | 0.02 | 0.5 | 26 | 0 | 0 | 18 |
| Cauliflower | B | 100 | 0.17 | 36 | 1.9 | 5.7 | 0.1 | 25 | 0.3 | 0.7 | 12 | 57 | 55 | 0.03 | 0.08 | 0.5 | 57 | 0 | 15 | 41 |
| Strawberry | C | 100 | 0.18 | 45 | 0.8 | 8.3 | 0.5 | 21 | 0.2 | 0.5 | 13 | 26 | 67 | 0.03 | 0.03 | 0.4 | 22 | 0 | 4 | 2 |
| Beans. dry. red | T | 100 | 0.28 | 392 | 22.9 | 59.7 | 1.3 | 120 | 3.5 | 5.3 | 156 | 418 | 2 | 0.69 | 0.2 | 1.5 | 604 | 0 | 0 | 6 |
| Beans. red | B | 100 | 0.38 | 166 | 10.5 | 29 | 0.4 | 67 | 2.83 | 3.3 | 147 | 220 | 16 | 0.39 | 0.08 | 1.4 | 521 | 0 | 4 | 0.95 |
| Salty cookies | A | 100 | 0.42 | 424 | 9.6 | 72.9 | 9.7 | 38 | 0.7 | 5.6 | 23 | 145 | 0 | 0.12 | 0.37 | 6.2 | 134 | 0.06 | 1 | 944 |
| Chickpeas | T | 100 | 0.21 | 393 | 19.9 | 58.8 | 5.5 | 120 | 3.1 | 6.4 | 127 | 300 | 4 | 0.48 | 0.22 | 1.4 | 557 | 0 | 3 | 30 |
| Soursop | C | 100 | 0.22 | 33 | 0.6 | 6.8 | 0.2 | 11 | 15.7 | 0.2 | 5 | 12 | 0 | 0.03 | 0.06 | 1.1 | 14 | 0 | 1 | 9 |
| Guava Pear | C | 100 | 0.07 | 78 | 0.9 | 15.1 | 0.4 | 16 | 0.2 | 0.3 | 14 | 26 | 214 | 0.04 | 0.04 | 1.1 | 49 | 0 | 31 | 3 |
| Strike bean | B | 100 | 0.10 | 43 | 2.1 | 7.2 | 0 | 40 | 0.2 | 1 | 22 | 39 | 10 | 0.06 | 0.1 | 0.7 | 34 | 0 | 33 | 6 |
| Wheat flour | A | 100 | 0.12 | 360 | 12.5 | 72.3 | 1.7 | 15 | 1 | 4.3 | 26 | 107 | 0 | 0.98 | 0.47 | 6.1 | 216 | 0 | 0 | 2 |
| Precooked corn flour | A | 100 | 0.12 | 380 | 9.1 | 73.9 | 3.7 | 4 | 1.3 | 2.7 | 44 | 248 | 0 | 0.3 | 0.12 | 2.7 | 35 | 0 | 0 | 1 |
| Egg | J | 100 | 0.31 | 149 | 12.6 | 0.3 | 10.8 | 53 | 1.4 | 1.7 | 12 | 197 | 0 | 0.07 | 0.49 | 0.1 | 48 | 1.29 | 177 | 139 |
| Pasteurized milk | G | 100 | 0.11 | 55 | 3.2 | 3.4 | 3.2 | 120 | 0.4 | 0 | 10 | 95 | 0 | 0.04 | 0.18 | 0.1 | 5 | 0.45 | 39 | 42 |
| Batavia lettuce | B | 100 | 0.17 | 18 | 0.8 | 2.6 | 0.1 | 21 | 0.3 | 0.9 | 6 | 27 | 3 | 0.06 | 0.04 | 0.4 | 53 | 0 | 10 | 6 |
| Lentil | T | 100 | 0.20 | 387 | 23.1 | 61 | 0.9 | 51 | 3.6 | 7.2 | 83 | 370 | 3 | 0.5 | 0.18 | 1.8 | 172 | 0 | 2 | 27 |
| Common lemons | C | 100 | 0.36 | 44 | 0.3 | 9.3 | 0.3 | 19 | 0.1 | 0.5 | 8 | 15 | 42 | 0.05 | 0.01 | 0.1 | 11 | 0 | 1 | 4 |
| Tahití lemons | C | 100 | 0.28 | 44 | 0.3 | 9.3 | 0.3 | 19 | 0.1 | 0.5 | 8 | 15 | 42 | 0.05 | 0.01 | 0.1 | 11 | 0 | 1 | 4 |
| Tuna loins | E | 100 | 0.73 | 211 | 25.5 | 0 | 12.1 | 8 | 0.5 | 1.5 | 34 | 250 | 0 | 0.02 | 0.08 | 12.3 | 5 | 2.2 | 5 | 391 |
| Naranjilla (lulo) | C | 100 | 0.26 | 48 | 0.9 | 8.9 | 0.1 | 10 | 0.1 | 0.6 | 19 | 15 | 20 | 0.04 | 0.04 | 0.1 | 7 | 0 | 37 | 0 |
| Tangerine | C | 100 | 0.14 | 54 | 0.9 | 11.4 | 0.1 | 35 | 0.2 | 0.3 | 12 | 21 | 24 | 0.06 | 0.03 | 0.2 | 18 | 0 | 35 | 2 |
| Common Mango | C | 100 | 0.24 | 79 | 0.6 | 17.1 | 0 | 15 | 0.1 | 0.5 | 24 | 21 | 48 | 0.12 | 0.12 | 0.5 | 39 | 0 | 140 | 4 |
| Tommy Atkins mango | C | 100 | 0.41 | 194 | 0.4 | 46.9 | 0.1 | 8 | 0.1 | 0.1 | 7 | 14 | 70 | 0.028 | 0.04 | 0.669 | 36.9 | 0 | 180 | 1 |
| Imported red apple | C | 100 | 0.27 | 72 | 0.3 | 16.5 | 0.2 | 16 | 0 | 0.3 | 5 | 10 | 10 | 0.05 | 0.02 | 0.2 | 1 | 0 | 3 | 2 |
| Gala (apple) | C | 100 | 0.26 | 72 | 0.3 | 16.5 | 0.2 | 16 | 0 | 0.3 | 5 | 10 | 10 | 0.05 | 0.02 | 0.2 | 1 | 0 | 3 | 2 |
| Imported green apple | C | 100 | 0.28 | 72 | 0.3 | 16.5 | 0.2 | 16 | 0 | 0.3 | 5 | 10 | 10 | 0.05 | 0.02 | 0.2 | 1 | 0 | 3 | 2 |
| Passion fruit | C | 100 | 0.45 | 60 | 1.5 | 12.4 | 0.5 | 9 | 0.1 | 1.7 | 25 | 21 | 20 | 0.01 | 0.17 | 0.8 | 8 | 0 | 173 | 6 |
| Melon | C | 100 | 0.22 | 25 | 0.7 | 5 | 0 | 11 | 0.1 | 0.4 | 7 | 17 | 0 | 0.04 | 0.02 | 0.6 | 21 | 0 | 68 | 7 |
| Raspberry | C | 100 | 0.13 | 74 | 1 | 14.6 | 0.1 | 42 | 0.5 | 1.7 | 20 | 10 | 18 | 0.22 | 0.05 | 0.9 | 25 | 0 | 0 | 1 |
| Sweet Orange | C | 100 | 0.07 | 41 | 0.7 | 8.8 | 0.3 | 33 | 0.5 | 1.3 | 213 | 20 | 27 | 0.35 | 0.16 | 0.2 | 30 | 0 | 2 | 2 |
| Unrefined whole cane sugar | K | 100 | 0.13 | 364 | 0.6 | 90.2 | 0.1 | 42 | 1.4 | 4.9 | 47 | 39 | 0 | 0.02 | 0.11 | 0.3 | 1 | 0 | 0 | 39 |
| Potato (*Betina)* | B | 100 | 0.04 | 89 | 2.1 | 18.9 | 0.1 | 12 | 0.3 | 0.8 | 23 | 57 | 20 | 0.08 | 0.03 | 1.1 | 16 | 0 | 0 | 6 |
| Potato (*Capira)* | B | 100 | 0.05 | 89 | 2.1 | 18.9 | 0.1 | 12 | 0.3 | 0.8 | 23 | 57 | 20 | 0.08 | 0.03 | 1.1 | 16 | 0 | 0 | 6 |
| Yellow creole potato (clean) | B | 100 | 0.07 | 96 | 2.2 | 21.1 | 0.1 | 11 | 0.29 | 1 | 23 | 36 | 32 | 0.11 | 0.03 | 2.4 | 16 | 0 | 2 | 18 |
| Yellow creole potato (dirty) | B | 100 | 0.07 | 96 | 2.2 | 21.1 | 0.1 | 11 | 0.29 | 1 | 23 | 36 | 32 | 0.11 | 0.03 | 2.4 | 16 | 0 | 2 | 18 |
| Potato (*Brown)* | B | 100 | 0.05 | 89 | 2.1 | 18.9 | 0.1 | 12 | 0.3 | 0.8 | 23 | 57 | 20 | 0.08 | 0.03 | 1.1 | 16 | 0 | 0 | 6 |
| Potato (*Suprema)* | B | 100 | 0.05 | 89 | 2.1 | 18.9 | 0.1 | 12 | 0.3 | 0.8 | 23 | 57 | 20 | 0.08 | 0.03 | 1.1 | 16 | 0 | 0 | 6 |
| Potato (*Unica)* | B | 100 | 0.04 | 89 | 2.1 | 18.9 | 0.1 | 12 | 0.3 | 0.8 | 23 | 57 | 20 | 0.08 | 0.03 | 1.1 | 16 | 0 | 0 | 6 |
| Papaya | C | 100 | 0.11 | 40 | 0.5 | 8.2 | 0.1 | 24 | 0.1 | 0.3 | 23 | 9 | 62 | 0.03 | 0.03 | 0.3 | 37 | 0 | 235 | 5 |
| Spaghetti | A | 100 | 0.20 | 371 | 13 | 74.7 | 1.5 | 25 | 1.3 | 3.9 | 53 | 120 | 0 | 0.95 | 0.39 | 7.1 | 237 | 0 | 0 | 6 |
| Watermelon | C | 100 | 0.19 | 17 | 0.4 | 3.7 | 0 | 4 | 0.1 | 0.3 | 10 | 5 | 7 | 0.02 | 0.01 | 0.1 | 3 | 0 | 30 | 1 |
| Chicken Breast | F | 100 | 0.40 | 166 | 20.7 | 0.1 | 9.2 | 11 | 0.8 | 0.7 | 25 | 174 | 0 | 0.07 | 0.09 | 9.9 | 4 | 0.34 | 24 | 63 |
| Cucumber | B | 100 | 0.08 | 14 | 0.5 | 2.3 | 0.1 | 18 | 0.1 | 0.3 | 12 | 22 | 8 | 0.02 | 0.02 | 0.1 | 14 | 0 | 2 | 2 |
| Imported pear | C | 100 | 0.25 | 63 | 0.3 | 12.9 | 0.2 | 8 | 0.1 | 0.4 | 5 | 9 | 2 | 0.07 | 0.04 | 0.2 | 7 | 0 | 0 | 1 |
| Chicken leg | F | 100 | 0.37 | 182 | 18.1 | 0.1 | 12.1 | 10 | 1.8 | 1 | 21 | 149 | 3 | 0.07 | 0.16 | 5.3 | 6 | 0.56 | 30 | 79 |
| Peppers | B | 100 | 0.09 | 35 | 1 | 6.3 | 0.3 | 7 | 0.2 | 0.4 | 12 | 24 | 160 | 0.05 | 0.07 | 1.1 | 35 | 0 | 184 | 2 |
| Pineapple gold | C | 100 | 0.12 | 56 | 0.6 | 12.4 | 0.1 | 16 | 0.2 | 0.5 | 12 | 9 | 23 | 0.06 | 0.05 | 0.3 | 16 | 0 | 8 | 3 |
| Ripe plantain | B | 100 | 0.10 | 132 | 1.1 | 30.3 | 0.2 | 3 | 0.1 | 0.5 | 37 | 36 | 15 | 0.07 | 0.05 | 0.5 | 22 | 0 | 54 | 4 |
| Whole chicken with viscera | F | 100 | 0.39 | 167 | 18.1 | 0.4 | 10.3 | 12 | 1.5 | 0.7 | 24 | 200 | 2 | 0.08 | 0.16 | 6.6 | 5 | 0.34 | 16 | 70 |
| Whole chicken without viscera | F | 100 | 0.50 | 167 | 18.1 | 0.4 | 10.3 | 12 | 1.5 | 0.7 | 24 | 200 | 2 | 0.08 | 0.16 | 6.6 | 5 | 0.34 | 16 | 70 |
| Cheese curd | G | 100 | 0.40 | 207 | 15.2 | 2 | 15.4 | 490 | 0.5 | 1.5 | 16 | 270 | 0 | 0.02 | 0.46 | 0.8 | 5 | 0.3 | 192 | 64 |
| Beetroot | B | 100 | 0.20 | 53 | 1.4 | 10.3 | 0.1 | 16 | 0.4 | 0.8 | 23 | 40 | 6 | 0.03 | 0.04 | 0.3 | 109 | 0 | 2 | 71 |
| Sardines | E | 100 | 0.57 | 225 | 24.6 | 0 | 14.1 | 350 | 1.6 | 2.9 | 40 | 430 | 0 | 0.08 | 0.29 | 6.7 | 8 | 8.94 | 28 | 450 |
| Whole red frozen tilapia fish | E | 100 | 0.81 | 96 | 20.1 | 0 | 1.7 | 10 | 0.3 | 0.6 | 27 | 170 | 0 | 0.04 | 0.06 | 3.9 | 24 | 1.59 | 0 | 52 |
| Frozen fillet tilapia fish | E | 100 | 0.58 | 85 | 18.3 | 0.1 | 1.3 | 27 | 0.3 | 0.8 | 24 | 186 | 0 | 0.06 | 0.07 | 1.3 | 12 | 1.2 | 17 | 75 |
| Tomato | B | 100 | 0.11 | 23 | 0.9 | 4.1 | 0.1 | 9 | 0.1 | 0.5 | 10 | 24 | 20 | 0.05 | 0.05 | 0.6 | 23 | 0 | 62 | 6 |
| Long shelf-life tomato | B | 100 | 0.19 | 23 | 0.9 | 4.1 | 0.1 | 9 | 0.1 | 0.5 | 10 | 24 | 20 | 0.05 | 0.05 | 0.6 | 23 | 0 | 62 | 6 |
| Fox grape | C | 100 | 0.10 | 51 | 0.4 | 12.1 | 0 | 8 | 0.1 | 0.4 | 4 | 10 | 14 | 0.03 | 0.01 | 0.1 | 27 | 0 | 7 | 9 |
| Red globe grape | C | 100 | 0.34 | 38 | 0.5 | 8.6 | 0 | 6 | 0.1 | 0.4 | 20 | 20 | 5 | 0.03 | 0.01 | 0.1 | 9 | 0 | 7 | 2 |
| Cassava | B | 100 | 0.17 | 159 | 0.9 | 37.4 | 0.3 | 16 | 0.3 | 0.3 | 21 | 30 | 30 | 0.04 | 0.03 | 0.8 | 27 | 0 | 1 | 14 |
| Carrot | B | 100 | 0.05 | 47 | 0.7 | 9.5 | 0.1 | 27 | 0.2 | 0.4 | 10 | 35 | 3 | 0.04 | 0.04 | 0.4 | 22 | 0 | 1318 | 35 |

**Table S2.** Food exchanges adjusted by caloric intake and Estimated Energy Requirements

|  | Group | Cereals. roots. tubers and plantains | Fruits and vegetables | Milk and dairy products | Meat. eggs. leguminous. nuts and seeds | Oils and fats | Sugars |
| --- | --- | --- | --- | --- | --- | --- | --- |
| Women | [1 – 4 years) | 4.59 | 1.96 | 1.96 | 4.40 | 2.93 | 0.98 |
|  | [4 – 9 years) | 4.37 | 2.62 | 2.62 | 5.53 | 3.49 | 1.75 |
|  | [9 – 14 years) | 6.05 | 3.85 | 3.85 | 6.60 | 4.40 | 3.30 |
|  | [14 – 19 years) | 8.55 | 5.70 | 4.99 | 9.26 | 5.70 | 4.27 |
|  | [19 – 31 years) | 6.34 | 4.23 | 3.70 | 6.87 | 4.23 | 3.17 |
|  | [31 – 51 years) | 5.98 | 3.98 | 3.49 | 6.47 | 3.98 | 2.99 |
|  | [51 – 70 years) | 5.40 | 3.60 | 3.15 | 5.85 | 3.60 | 2.70 |
|  | ≥70 years | 4.83 | 3.08 | 3.08 | 5.27 | 4.39 | 2.64 |
|  |  |  |  |  |  |  |  |
| Pregnancy | > 18 years | 9.36 | 3.74 | 3.74 | 8.48 | 3.62 | 4.37 |
|  | [19 – 31 years) | 9.88 | 3.70 | 4.94 | 8.64 | 2.96 | 2.47 |
|  | [31 – 51 years) | 9.42 | 3.53 | 4.71 | 8.24 | 2.83 | 2.35 |
|  |  |  |  |  |  |  |  |
| Lactation | > 18 years | 9.58 | 3.59 | 4.19 | 8.38 | 3.47 | 4.19 |
|  | [19 – 31 years) | 9.89 | 3.71 | 4.94 | 8.65 | 3.58 | 2.47 |
|  | [31 – 51 years) | 9.43 | 3.54 | 4.72 | 8.25 | 3.42 | 2.36 |
|  |  |  |  |  |  |  |  |
| Men | [1 – 4 years) | 3.42 | 1.96 | 1.96 | 4.40 | 2.93 | 0.98 |
|  | [4 – 9 years) | 4.78 | 2.87 | 2.87 | 6.05 | 3.82 | 1.91 |
|  | [9 – 14 years) | 6.05 | 3.85 | 3.85 | 6.60 | 4.40 | 3.30 |
|  | [14 – 19 years) | 9.11 | 5.89 | 5.36 | 8.57 | 5.36 | 3.75 |
|  | [19 – 31 years) | 9.37 | 5.86 | 5.86 | 8.79 | 5.86 | 3.51 |
|  | [31 – 51 years) | 8.86 | 5.54 | 5.54 | 8.30 | 5.54 | 3.32 |
|  | [51 – 70 years) | 7.22 | 4.51 | 4.51 | 6.76 | 4.51 | 2.71 |
|  | ≥70 years | 6.02 | 3.83 | 3.83 | 6.56 | 5.47 | 3.28 |
| **Source:** Own calculations based on information from Food-based dietary guidelines for Colombian population (Guías Alimentarias Basadas en Alimentos para la población colombiana [GABA]). | | | | | | | |

**Table S3.** Cost of Caloric Adequacy (CoCA) per day by population group (USD [COP])

|  | **Women** | | **Pregnancy** | | **Lactating** | | **Men** | |
| --- | --- | --- | --- | --- | --- | --- | --- | --- |
|  | **Rice (g)** | **Cost per day** | **Rice (g)** | **Cost per day** | **Rice (g)** | **Cost per day** | **Rice (g)** | **Cost per day** |
| [1 – 4 years) | 314.4 | 0.35 (1323.2) | - | - | - | - | 314.4 | 0.35 (1323.2) |
| [4 – 9 years) | 410.8 | 0.46 (1728.5) | - | - | - | - | 449.6 | 0.5 (1891.8) |
| [9 – 14 years) | 569.4 | 0.63 (2396.1) | - | - | - | - | 569.4 | 0.63 (2396.1) |
| [14 – 19 years) | 779 | 0.87 (3278.2) | 714 | 0.79 (3004.5) | 722.5 | 0.8 (3040.2) | 777.3 | 0.86 (3271) |
| [19 – 31 years) | 577.6 | 0.64 (2430.5) | 712.2 | 0.79 (2996.8) | 720.7 | 0.8 (3032.5) | 803.2 | 0.89 (3379.8) |
| [31 – 51 years) | 544.6 | 0.61 (2291.5) | 679.1 | 0.76 (2857.8) | 687.6 | 0.76 (2893.5) | 759 | 0.84 (3193.8) |
| [51 – 70 years) | 492 | 0.55 (2070.5) | - | - | - | - | 618.3 | 0.69 (2602) |
| ≥70 years | 463.5 | 0.52 (1950.5) | - | - | - | - | 576.9 | 0.64 (2427.7) |
| **Source:** Own calculations based on the information of the study. | | | | | | | | |

**Table S4.** Cost of nutrient adequacy (CoNA) per day and per 1000 kcal by population group (USD [COP])

|  | **Women** | | **Pregnancy** | | **Lactating** | | **Men** | |
| --- | --- | --- | --- | --- | --- | --- | --- | --- |
|  | **Cost per day** | **Cost per 1000**  **kcal** | **Cost per day** | **Cost per 1000**  **kcal** | **Cost per day** | **Cost per 1000**  **kcal** | **Cost per day** | **Cost per 1000**  **kcal** |
| [1 – 4 years) | 0.68 (2586.8) | 0.62 (2330.45) | - | - | - | - | 0.94 (3560.7) | 0.85 (3207.84) |
| [4 – 9 years) | 1.25 (4732.9) | 0.86 (3264.07) | - | - | - | - | 1.29 (4888.5) | 0.81 (3080.34) |
| [9 – 14 years) | 1.68 (6374.3) | 0.84 (3171.29) | - | - | - | - | 1.68 (6375) | 0.84 (3171.64) |
| [14 – 19 years) | 1.87 (7088.3) | 0.68 (2577.56) | 2.07 (7845.5) | 0.82 (3112.8) | 1.87 (7080.4) | 0.73 (2776.19) | 1.91 (7228.2) | 0.7 (2634.18) |
| [19 – 31 years) | 1.51 (5734) | 0.74 (2812.26) | 1.8 (6819) | 0.72 (2712.49) | 1.69 (6378.4) | 0.66 (2507.3) | 1.82 (6892.7) | 0.64 (2431.07) |
| [31 – 51 years) | 1.49 (5623.6) | 0.77 (2925.41) | 1.79 (6792.8) | 0.75 (2833.49) | 1.64 (6223.2) | 0.68 (2563.8) | 1.75 (6608.6) | 0.65 (2466.6) |
| [51 – 70 years) | 1.5 (5692.3) | 0.87 (3277.22) | - | - | - | - | 1.65 (6251.1) | 0.76 (2863.84) |
| ≥70 years | 1.45 (5491.2) | 0.89 (3356.07) | - | - | - | - | 1.54 (5832.3) | 0.76 (2863.86) |
| **Source:** Own calculations based on the information of the study. | | | | | | | | |

**Table S5.** Cost of Recommended diet (CoRD) per day by population group (USD [COP])

|  | **Women** | **Pregnancy** | **Lactating** | **Men** |
| --- | --- | --- | --- | --- |
| [1 – 4 years) | 1.27 (4790) | - | - | 1.17 (4440.9) |
| [4 – 9 years) | 1.52 (5759.9) | - | - | 1.67 (6304.1) |
| [9 – 14 years) | 2.06 (7809.8) | - | - | 2.06 (7809.8) |
| [14 – 19 years) | 2.83 (10697.4) | 2.5 (9455.5) | 2.57 (9720.1) | 2.85 (10804.3) |
| [19 – 31 years) | 2.1 (7931.4) | 2.7 (10205.6) | 2.71 (10254.8) | 2.98 (11284.4) |
| [31 – 51 years) | 1.98 (7477.8) | 2.57 (9732.2) | 2.59 (9784.8) | 2.82 (10663.4) |
| [51 – 70 years) | 1.79 (6756.6) | - | - | 2.3 (8687.5) |
| ≥70 years | 1.66 (6300.2) | - | - | 2.07 (7841.7) |
| **Source:** Own calculations based on the information of the study. | | | | |

**Table S6.** Model validation for Cost of Recommended Diet (CoRD)

|  | | **Men** | | | | | | | |
| --- | --- | --- | --- | --- | --- | --- | --- | --- | --- |
|  | | **[1 - 4)** | **[4 - 9)** | **[9 - 14)** | **[14 - 19)** | **[19 - 31)** | **[31 - 51)** | **[51 - 70)** | **≥70** |
| **Energy (Kcal)** | **Content** | 1117.4 | 1601.7 | 2020.6 | 2745.9 | 2831.5 | 2675.6 | 2179.9 | 2058.5 |
|  | **LB** | 1110.0 | 1587.0 | 2010.0 | 2744.0 | 2835.3 | 2679.2 | 2182.8 | 2036.5 |
|  | **UP** | - | - | - | - | - | - | - | - |
| **Protein (g)** | **Content** | 42.0 | 59.1 | 70.7 | 96.7 | 101.3 | 95.7 | 78.0 | 70.4 |
|  | **LB** | 27.8 | 39.7 | 50.3 | 68.6 | 99.2 | 93.8 | 76.4 | 71.3 |
|  | **UP** | 55.5 | 79.4 | 100.5 | 137.2 | 141.8 | 134.0 | 109.1 | 101.8 |
| **Fats (g)** | **Content** | 42.1 | 58.0 | 69.1 | 90.6 | 97.0 | 91.6 | 74.7 | 74.1 |
|  | **LB** | 37.0 | 44.1 | 55.8 | 76.2 | 63.0 | 59.5 | 48.5 | 45.3 |
|  | **UP** | 49.3 | 61.7 | 78.2 | 106.7 | 110.3 | 104.2 | 84.9 | 79.2 |
| **CHO (g)** | **Content** | 136.1 | 201.6 | 267.8 | 370.2 | 372.4 | 351.9 | 286.7 | 266.5 |
|  | **LB** | 138.8 | 198.4 | 251.3 | 343.0 | 354.4 | 334.9 | 272.8 | 254.6 |
|  | **UP** | 180.4 | 257.9 | 326.6 | 445.9 | 460.7 | 435.4 | 354.7 | 330.9 |
| **Vitamin C (mg)** | **Content** | 117.0 | 168.4 | 221.2 | 336.5 | 338.5 | 319.9 | 260.6 | 220.0 |
|  | **LB** | 13.0 | 22.0 | 39.0 | 63.0 | 75.0 | 75.0 | 75.0 | 75.0 |
|  | **UP** | 400.0 | 650.0 | 1200.0 | 1800.0 | 2000.0 | 2000.0 | 2000.0 | 2000.0 |
| **Folate (mcg)** | **Content** | 207.5 | 289.4 | 337.7 | 467.8 | 477.5 | 451.3 | 367.6 | 336.0 |
|  | **LB** | 120.0 | 160.0 | 250.0 | 330.0 | 320.0 | 320.0 | 320.0 | 320.0 |
|  | **UP** | 300.0 | 400.0 | 600.0 | 800.0 | 1000.0 | 1000.0 | 1000.0 | 1000.0 |
| **Vitamin A (EAR)** | **Content** | 1181.4 | 1726.6 | 2306.6 | 3483.3 | 3510.3 | 3317.1 | 2702.5 | 2294.9 |
|  | **LB** | 210.0 | 275.0 | 445.0 | 630.0 | 625.0 | 625.0 | 625.0 | 625.0 |
|  | **UP** | 600.0 | 900.0 | 1700.0 | 2800.0 | 3000.0 | 3000.0 | 3000.0 | 3000.0 |
| **Thiamine (mg)** | **Content** | 1.0 | 1.4 | 1.6 | 2.2 | 2.2 | 2.1 | 1.7 | 1.6 |
|  | **LB** | 0.4 | 0.5 | 0.7 | 1.0 | 1.0 | 1.0 | 1.0 | 1.0 |
|  | **UP** | - | - | - | - | - | - | - | - |
| **Riboflavin (mg)** | **Content** | 0.8 | 1.1 | 1.4 | 1.9 | 2.0 | 1.9 | 1.6 | 1.4 |
|  | **LB** | 0.4 | 0.5 | 0.8 | 1.1 | 1.1 | 1.1 | 1.1 | 1.1 |
|  | **UP** | - | - | - | - | - | - | - | - |
| **Niacin (mg)** | **Content** | 11.5 | 16.1 | 19.4 | 27.6 | 28.4 | 26.8 | 21.9 | 19.3 |
|  | **LB** | 5.0 | 6.0 | 9.0 | 12.0 | 12.0 | 12.0 | 12.0 | 12.0 |
|  | **UP** | 10.0 | 15.0 | 20.0 | 30.0 | 35.0 | 35.0 | 35.0 | 35.0 |
| **Vitamin B12 (mcg)** | **Content** | 0.8 | 1.1 | 1.3 | 1.7 | 1.8 | 1.7 | 1.4 | 1.3 |
|  | **LB** | 0.7 | 1.0 | 1.5 | 2.0 | 2.0 | 2.0 | 2.0 | 2.0 |
|  | **UP** | - | - | - | - | - | - | - | - |
| **Magnesium (mg)** | **Content** | 151.3 | 212.1 | 255.3 | 360.8 | 370.9 | 350.4 | 285.5 | 254.1 |
|  | **LB** | 65.0 | 110.0 | 200.0 | 340.0 | 330.0 | 350.0 | 350.0 | 360.0 |
|  | **UP** | 65.0 | 110.0 | 350.0 | 350.0 | 350.0 | 350.0 | 350.0 | 350.0 |
| **Phosphorus (mg)** | **Content** | 692.4 | 978.5 | 1199.3 | 1672.6 | 1753.7 | 1657.2 | 1350.1 | 1193.3 |
|  | **LB** | 380.0 | 405.0 | 1055.0 | 1055.0 | 580.0 | 580.0 | 580.0 | 580.0 |
|  | **UP** | 3000.0 | 3000.0 | 4000.0 | 4000.0 | 4000.0 | 4000.0 | 4000.0 | 3000.0 |
| **Sodium (mg)** | **Content** | 139.1 | 197.3 | 241.0 | 331.7 | 348.7 | 329.5 | 268.4 | 239.8 |
|  | **LB** | 1000.0 | 1200.0 | 1500.0 | 1500.0 | 1500.0 | 1500.0 | 1300.0 | 1200.0 |
|  | **UP** | 1500.0 | 1900.0 | 2200.0 | 2300.0 | 2300.0 | 2300.0 | 2300.0 | 2300.0 |
| **Calcium (mg)** | **Content** | 491.9 | 715.2 | 943.5 | 1322.0 | 1427.9 | 1349.3 | 1099.3 | 938.7 |
|  | **LB** | 500.0 | 800.0 | 1100.0 | 1100.0 | 800.0 | 800.0 | 1000.0 | 1000.0 |
|  | **UP** | 2500.0 | 2500.0 | 3000.0 | 3000.0 | 2500.0 | 2500.0 | 2000.0 | 2000.0 |
| **Iron (mg)** | **Content** | 6.8 | 9.6 | 11.7 | 16.5 | 17.1 | 16.2 | 13.2 | 11.7 |
|  | **LB** | 4.5 | 6.2 | 8.9 | 11.6 | 9.0 | 9.0 | 9.0 | 9.0 |
|  | **UP** | 40.0 | 40.0 | 40.0 | 45.0 | 45.0 | 45.0 | 45.0 | 45.0 |
| **Zinc (mg)** | **Content** | 4.2 | 5.8 | 6.7 | 9.2 | 9.5 | 9.0 | 7.3 | 6.7 |
|  | **LB** | 2.5 | 3.0 | 5.0 | 11.0 | 12.0 | 12.0 | 12.0 | 12.0 |
|  | **UP** | 7.0 | 12.0 | 25.0 | 35.0 | 40.0 | 40.0 | 40.0 | 40.0 |

**(a)**

|  | | **Women** | | | | | | | | **Pregnancy** | | | **Lactating** | | |
| --- | --- | --- | --- | --- | --- | --- | --- | --- | --- | --- | --- | --- | --- | --- | --- |
|  | | **1 - 3** | **4 - 8** | **9 - 13** | **14 - 18** | **19 - 30** | **31 - 50** | **51 - 70** | **>70** | **< 18** | **19 - 30** | **31 - 50** | **< 18** | **19 - 30** | **31 - 50** |
| **Energy (Kcal)** | **Content** | 1223.2 | 1463.4 | 2020.6 | 2768.4 | 2052.6 | 1935.2 | 1748.6 | 1653.8 | 2510.4 | 2489.6 | 2374.1 | 2534.0 | 2518.9 | 2403.5 |
|  | **LB** | 1110.0 | 1450.0 | 2010.0 | 2750.0 | 2038.9 | 1922.3 | 1736.9 | 1636.2 | 2520.4 | 2513.9 | 2397.3 | 2550.4 | 2543.9 | 2427.3 |
|  | **UP** | - | - | - | - | - | - | - | - | - | - | - | - | - | - |
| **Protein (g)** | **Content** | 43.8 | 54.0 | 70.7 | 97.0 | 71.9 | 67.8 | 61.2 | 56.5 | 85.1 | 94.2 | 89.9 | 87.6 | 94.3 | 90.0 |
|  | **LB** | 27.8 | 36.3 | 50.3 | 68.8 | 71.4 | 67.3 | 60.8 | 57.3 | 88.2 | 88.0 | 83.9 | 89.3 | 89.0 | 85.0 |
|  | **UP** | 55.5 | 72.5 | 100.5 | 137.5 | 101.9 | 96.1 | 86.8 | 81.8 | 126.0 | 125.7 | 119.9 | 127.5 | 127.2 | 121.4 |
| **Fats (g)** | **Content** | 42.2 | 53.0 | 69.1 | 92.1 | 68.3 | 64.4 | 58.2 | 59.5 | 71.2 | 76.3 | 72.7 | 73.0 | 79.3 | 75.7 |
|  | **LB** | 37.0 | 40.3 | 55.8 | 76.4 | 45.3 | 42.7 | 38.6 | 36.4 | 70.0 | 55.9 | 53.3 | 70.8 | 56.5 | 53.9 |
|  | **UP** | 49.3 | 56.4 | 78.2 | 106.9 | 79.3 | 74.8 | 67.5 | 63.6 | 98.0 | 97.8 | 93.2 | 99.2 | 98.9 | 94.4 |
| **CHO (g)** | **Content** | 159.9 | 184.2 | 267.8 | 372.1 | 275.9 | 260.1 | 235.0 | 214.1 | 368.3 | 342.3 | 326.4 | 367.7 | 342.6 | 326.9 |
|  | **LB** | 138.8 | 181.3 | 251.3 | 343.8 | 254.9 | 240.3 | 217.1 | 204.5 | 315.0 | 314.2 | 299.7 | 318.8 | 318.0 | 303.4 |
|  | **UP** | 180.4 | 235.6 | 326.6 | 446.9 | 331.3 | 312.4 | 282.3 | 265.9 | 409.6 | 408.5 | 389.6 | 414.4 | 413.4 | 394.4 |
| **Vitamin C (mg)** | **Content** | 131.5 | 153.9 | 221.2 | 322.4 | 239.1 | 225.4 | 203.6 | 176.8 | 258.6 | 263.6 | 251.4 | 255.6 | 263.9 | 251.8 |
|  | **LB** | 12.0 | 22.0 | 39.0 | 56.0 | 60.0 | 60.0 | 60.0 | 60.0 | 66.0 | 70.0 | 70.0 | 96.0 | 100.0 | 100.0 |
|  | **UP** | 400.0 | 650.0 | 1200.0 | 1800.0 | 2000.0 | 2000.0 | 2000.0 | 2000.0 | 1800.0 | 2000.0 | 2000.0 | 1800.0 | 2000.0 | 2000.0 |
| **Folate (mcg)** | **Content** | 219.8 | 264.4 | 337.7 | 478.9 | 355.1 | 334.8 | 302.5 | 270.0 | 428.4 | 440.7 | 420.2 | 426.0 | 441.1 | 420.8 |
|  | **LB** | 120.0 | 160.0 | 250.0 | 330.0 | 320.0 | 320.0 | 320.0 | 320.0 | 520.0 | 520.0 | 520.0 | 450.0 | 450.0 | 450.0 |
|  | **UP** | 300.0 | 400.0 | 600.0 | 800.0 | 1000.0 | 1000.0 | 1000.0 | 1000.0 | 800.0 | 1000.0 | 1000.0 | 800.0 | 1000.0 | 1000.0 |
| **Vitamin A (EAR)** | **Content** | 1199.5 | 1577.5 | 2306.6 | 3351.6 | 2485.0 | 2342.9 | 2116.9 | 1843.8 | 2303.5 | 2388.8 | 2278.0 | 2268.3 | 2390.9 | 2281.3 |
|  | **LB** | 210.0 | 275.0 | 420.0 | 485.0 | 500.0 | 500.0 | 500.0 | 500.0 | 530.0 | 550.0 | 550.0 | 885.0 | 900.0 | 900.0 |
|  | **UP** | 600.0 | 900.0 | 1700.0 | 2800.0 | 3000.0 | 3000.0 | 3000.0 | 3000.0 | 2800.0 | 3000.0 | 3000.0 | 2800.0 | 3000.0 | 3000.0 |
| **Thiamine (mg)** | **Content** | 1.1 | 1.3 | 1.6 | 2.2 | 1.7 | 1.6 | 1.4 | 1.3 | 2.1 | 2.1 | 2.0 | 2.1 | 2.1 | 2.0 |
|  | **LB** | 0.4 | 0.5 | 0.7 | 0.9 | 0.9 | 0.9 | 0.9 | 0.9 | 1.2 | 1.2 | 1.2 | 1.2 | 1.2 | 1.2 |
|  | **UP** | - | - | - | - | - | - | - | - | - | - | - | - | - | - |
| **Riboflavin (mg)** | **Content** | 0.8 | 1.0 | 1.4 | 1.8 | 1.4 | 1.3 | 1.2 | 1.1 | 1.5 | 1.8 | 1.7 | 1.6 | 1.8 | 1.7 |
|  | **LB** | 0.4 | 0.5 | 0.8 | 0.9 | 0.9 | 0.9 | 0.9 | 0.9 | 1.2 | 1.2 | 1.2 | 1.3 | 1.3 | 1.3 |
|  | **UP** | - | - | - | - | - | - | - | - | - | - | - | - | - | - |
| **Niacin (mg)** | **Content** | 13.3 | 14.7 | 19.4 | 27.2 | 20.2 | 19.0 | 17.2 | 15.5 | 26.4 | 27.8 | 26.5 | 26.8 | 27.8 | 26.6 |
|  | **LB** | 5.0 | 6.0 | 9.0 | 11.0 | 11.0 | 11.0 | 11.0 | 11.0 | 14.0 | 14.0 | 14.0 | 13.0 | 13.0 | 13.0 |
|  | **UP** | 10.0 | 15.0 | 20.0 | 30.0 | 35.0 | 35.0 | 35.0 | 35.0 | 30.0 | 35.0 | 35.0 | 30.0 | 35.0 | 35.0 |
| **Vitamin B12 (mcg)** | **Content** | 0.8 | 1.0 | 1.3 | 1.8 | 1.3 | 1.2 | 1.1 | 1.0 | 1.5 | 1.7 | 1.6 | 1.6 | 1.7 | 1.6 |
|  | **LB** | 0.7 | 1.0 | 1.5 | 2.0 | 2.0 | 2.0 | 2.0 | 2.0 | 2.2 | 2.2 | 2.2 | 2.4 | 2.4 | 2.4 |
|  | **UP** | - | - | - | - | - | - | - | - | - | - | - | - | - | - |
| **Magnesium (mg)** | **Content** | 167.9 | 193.8 | 255.3 | 359.5 | 266.5 | 251.3 | 227.1 | 204.1 | 330.7 | 347.8 | 331.7 | 333.6 | 348.1 | 332.2 |
|  | **LB** | 65.0 | 110.0 | 200.0 | 300.0 | 255.0 | 265.0 | 265.0 | 265.0 | 335.0 | 290.0 | 300.0 | 300.0 | 255.0 | 265.0 |
|  | **UP** | 65.0 | 110.0 | 350.0 | 350.0 | 350.0 | 350.0 | 350.0 | 350.0 | 350.0 | 350.0 | 350.0 | 350.0 | 350.0 | 350.0 |
| **Phosphorus (mg)** | **Content** | 745.7 | 894.0 | 1199.3 | 1645.5 | 1220.0 | 1150.3 | 1039.3 | 958.7 | 1456.2 | 1623.7 | 1548.4 | 1507.1 | 1625.2 | 1550.7 |
|  | **LB** | 380.0 | 405.0 | 1055.0 | 1055.0 | 580.0 | 580.0 | 580.0 | 580.0 | 1055.0 | 580.0 | 580.0 | 1055.0 | 580.0 | 580.0 |
|  | **UP** | 3000.0 | 3000.0 | 4000.0 | 4000.0 | 4000.0 | 4000.0 | 4000.0 | 3000.0 | 3500.0 | 3500.0 | 3500.0 | 4000.0 | 4000.0 | 4000.0 |
| **Sodium (mg)** | **Content** | 142.0 | 180.2 | 241.0 | 329.4 | 244.3 | 230.3 | 208.1 | 192.7 | 274.0 | 309.3 | 295.0 | 284.0 | 309.6 | 295.4 |
|  | **LB** | 100.0 | 1200.0 | 1500.0 | 1500.0 | 1500.0 | 1500.0 | 1300.0 | 1200.0 | 1500.0 | 1500.0 | 1500.0 | 1500.0 | 1500.0 | 1500.0 |
|  | **UP** | 1500.0 | 1900.0 | 2200.0 | 2300.0 | 2300.0 | 2300.0 | 2300.0 | 2300.0 | 2300.0 | 2300.0 | 2300.0 | 2300.0 | 2300.0 | 2300.0 |
| **Calcium (mg)** | **Content** | 504.4 | 653.5 | 943.5 | 1243.9 | 922.3 | 869.5 | 785.7 | 754.2 | 971.3 | 1221.4 | 1164.7 | 1062.3 | 1222.5 | 1166.4 |
|  | **LB** | 500.0 | 800.0 | 1100.0 | 1100.0 | 800.0 | 800.0 | 1000.0 | 1000.0 | 1100.0 | 800.0 | 800.0 | 1100.0 | 800.0 | 800.0 |
|  | **UP** | 2500.0 | 2500.0 | 3000.0 | 3000.0 | 2500.0 | 2500.0 | 2000.0 | 2000.0 | 3000.0 | 2500.0 | 2500.0 | 3000.0 | 2500.0 | 2500.0 |
| **Iron (mg)** | **Content** | 7.5 | 8.8 | 11.7 | 16.3 | 12.1 | 11.4 | 10.3 | 9.4 | 14.8 | 15.9 | 15.2 | 15.1 | 15.9 | 15.2 |
|  | **LB** | 4.5 | 6.2 | 8.5 | 11.9 | 11.7 | 11.7 | 7.5 | 7.5 | 34.0 | 33.0 | 33.0 | 10.5 | 9.8 | 9.8 |
|  | **UP** | 40.0 | 40.0 | 40.0 | 45.0 | 45.0 | 45.0 | 45.0 | 45.0 | 45.0 | 45.0 | 45.0 | 45.0 | 45.0 | 45.0 |
| **Zinc (mg)** | **Content** | 4.4 | 5.3 | 6.7 | 9.4 | 7.0 | 6.6 | 5.9 | 5.4 | 8.6 | 9.0 | 8.6 | 8.6 | 9.0 | 8.6 |
|  | **LB** | 2.5 | 3.0 | 5.0 | 6.0 | 6.5 | 6.5 | 6.5 | 6.5 | 8.5 | 9.0 | 9.0 | 9.0 | 10.0 | 10.0 |
|  | **UP** | 7.0 | 12.0 | 25.0 | 35.0 | 40.0 | 40.0 | 40.0 | 40.0 | 35.0 | 40.0 | 40.0 | 35.0 | 40.0 | 40.0 |

**(b)**

Model validation for Cost of Recommended Diet (CoRD) by comparing diet nutrient content and nutrient requirements for (a) males and (b) females of all age groups. Content = Nutrient content of the Recommended Diet. LB = minimum level of intake needed for the nutrient. UP = maximum level of intake needed for the nutrient. **Source:** Own calculations based on the information of the study.

**Table S7.** Lower-bound and upper-bound estimates of affordability indicators for least-cost diets by per capita household income percentiles

| **Percentile** | **Lower-bound estimates** | | | | | |  | **Upper-bound estimates** | | | | | |
| --- | --- | --- | --- | --- | --- | --- | --- | --- | --- | --- | --- | --- | --- |
|  | **CoCA** | | **CoNA** | | **CoRD** | |  | **CoCA** | | **CoNA** | | **CoRD** | |
|  | **Rate (%)** | **Ratio** | **Rate (%)** | **Ratio** | **Rate (%)** | **Ratio** |  | **Rate (%)** | **Ratio** | **Rate (%)** | **Ratio** | **Rate (%)** | **Ratio** |
| **10** | 13,71 | 0,39 | 42,22 | 0,93 | 67,67 | 1,29 |  | 42,66 | 1,01 | 100,00 | 2,37 | 100,00 | 3,31 |
| **20** | 0,00 | 0,20 | 0,00 | 0,46 | 0,00 | 0,64 |  | 0,00 | 0,50 | 100,00 | 1,18 | 100,00 | 1,65 |
| **30** | 0,00 | 0,15 | 0,00 | 0,34 | 0,00 | 0,48 |  | 0,00 | 0,41 | 40,97 | 0,96 | 100,00 | 1,34 |
| **40** | 0,00 | 0,11 | 0,00 | 0,26 | 0,00 | 0,37 |  | 0,00 | 0,31 | 0,00 | 0,73 | 60,77 | 1,02 |
| **50** | 0,00 | 0,09 | 0,00 | 0,22 | 0,00 | 0,30 |  | 0,00 | 0,26 | 0,00 | 0,62 | 0,00 | 0,87 |
| **60** | 0,00 | 0,08 | 0,00 | 0,18 | 0,00 | 0,25 |  | 0,00 | 0,22 | 0,00 | 0,52 | 0,00 | 0,73 |
| **70** | 0,00 | 0,06 | 0,00 | 0,15 | 0,00 | 0,21 |  | 0,00 | 0,20 | 0,00 | 0,46 | 0,00 | 0,64 |
| **80** | 0,00 | 0,05 | 0,00 | 0,11 | 0,00 | 0,16 |  | 0,00 | 0,15 | 0,00 | 0,36 | 0,00 | 0,50 |
| **90** | 0,00 | 0,03 | 0,00 | 0,08 | 0,00 | 0,11 |  | 0,00 | 0,13 | 0,00 | 0,31 | 0,00 | 0,43 |
| **100** | 0,00 | 0,02 | 0,00 | 0,04 | 0,00 | 0,05 |  | 0,00 | 0,06 | 0,00 | 0,14 | 0,00 | 0,20 |
| Lower-bound estimates assume that household allocate 100% of their total income to food expenditure. Upper-bound estimates reflect varying proportions of income spent on food across income quintiles, with households in the first quintile allocating 39%, the second quintile 36%, the third quintile 35%, the fourth quintile 32%, and the fifth quintile 26%. CoCA = Cost of Caloric Adequacy. CoNA = Cost of Nutrient Adequacy. CoRD = Cost of a Recommended Diet. Rate = the proportion (%) of households unable to afford each least-cost diet. Ratio = estimated per capita minimum cost of a diet for the representative household relative to average per capita household expenditure on food. **Source:** Own calculations based on the information of the study. | | | | | | | | | | | | | |
